# Supplementary material for: 4-Azidocinnoline—Cinnoline-4-amine Pair as a New Fluorogenic and Fluorochromic Environment-Sensitive Probe
Source: Molecules. 2021 Dec 9;26(24):7460. doi: 10.3390/molecules26247460 (PMC8704291; doi:10.3390/molecules26247460)
Supplement: Supplementary file 1 [file molecules-26-07460-s001.zip › molecules-1485490-SI.pdf]

Supporting Information

4-Azidocinnoline – Cinnoline-4-amine Pair as a New Fluorogenic and Fluorochromic Environment-Sensitive Probe

Natalia A. Danilkina [1], Ekaterina V. Andrievskaya [2], Anna V. Vasileva [1], Anna L. Lyapunova [1], Andrey M. Rumyantsev [3], Andrey A. Kuzmin [4], Elena A. Bessonova [1] and Irina A. Balova [1,*]

[1] Institute of Chemistry, Saint Petersburg State University (SPbU), Universitetskaya nab. 7/9, 199034 Saint Petersburg, Russia; i.balova@spbu.ru
[2] Faculty of Chemistry, Belarusian State University, Leningradskaya str. 14, 220006 Minsk, Belarus; kateandrievskaya@yandex.by
[3] Department of Genetics and Biotechnology, Saint Petersburg State University (SPbU), Universitetskaya nab. 7/9, 199034 Saint Petersburg, Russia
[4] Institute of Cytology, Russian Academy of Sciences, St Petersburg, Tikhoretsky avenue 4, 194064, Saint Petersburg, Russia; a.kuzmin@incras.ru
* Correspondence: i.balova@spbu.ru; Tel.: +78124286733

Table of Contents

Copies of NMR Spectra and ESI HRMS……………………………….……S2

Details of HPLC Measurements and Copies of HPLC Chromatograms…S29

Details of QY Fluorescence Measurements……….…………………...S31

Experimental details for the synthesis of amines **7a,b**…………………...S32

References……………………………………………………………...S34

^{1}H NMR, acetone-d_6, 400 MHz

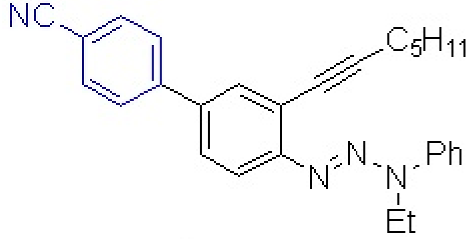

3

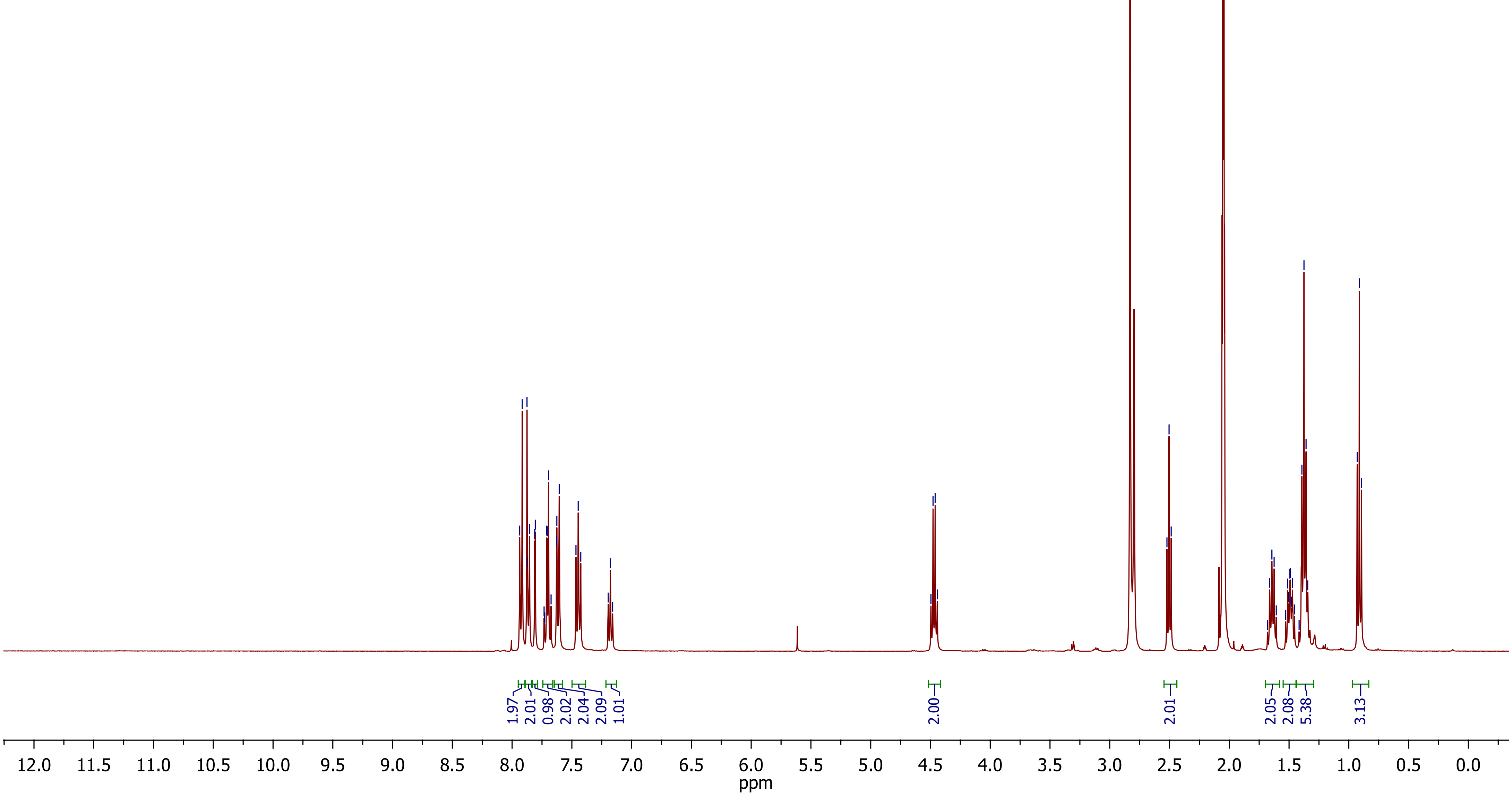

^{1}H NMR, acetone-d_6, 400 MHz

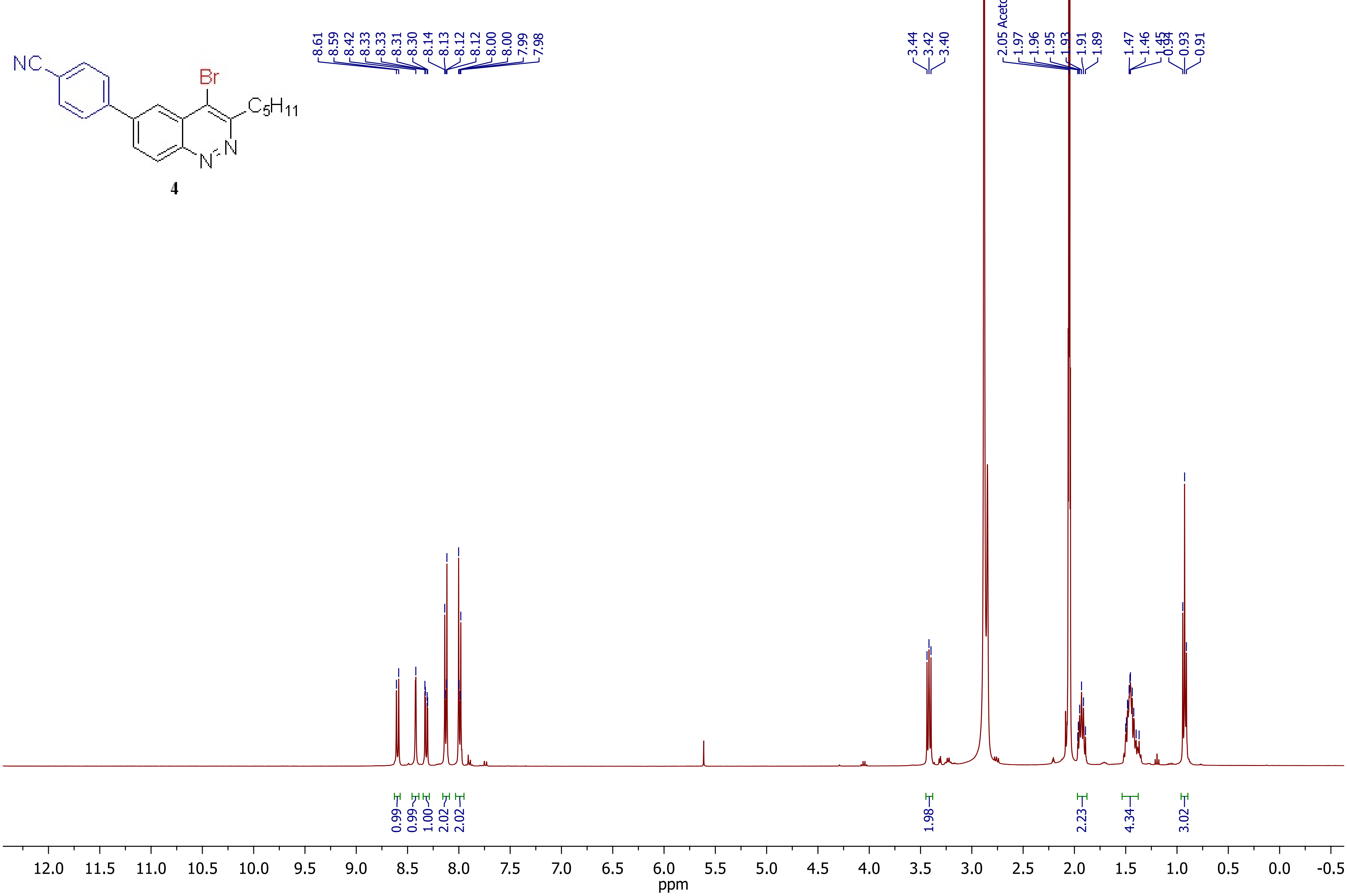

^{1}H NMR, acetone-d_6, 400 MHz

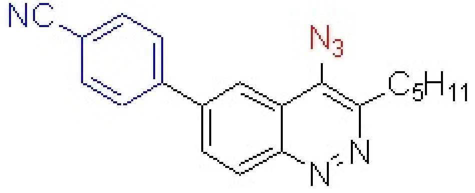

5

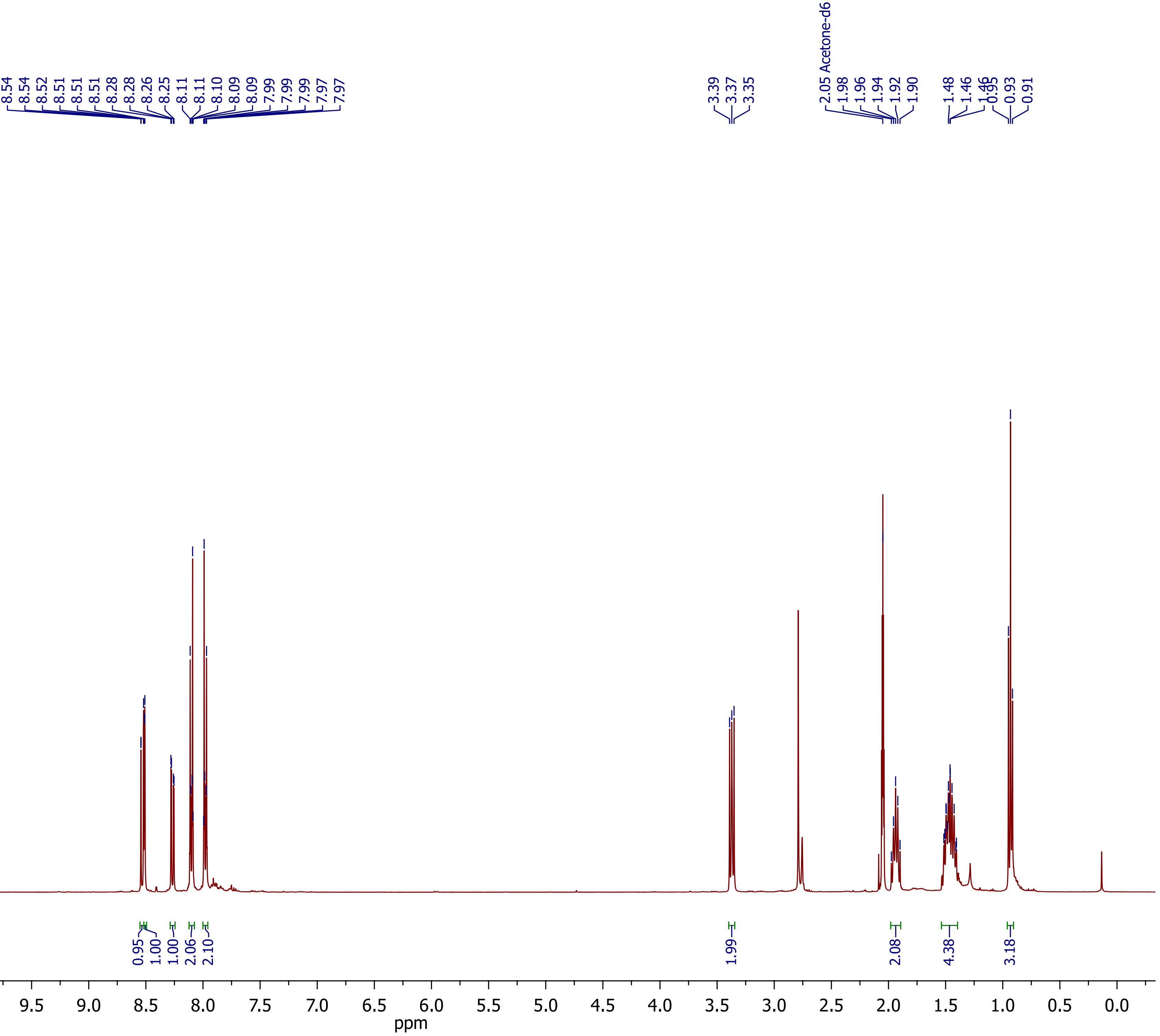

^{1}H NMR, DMSO-d_6, 400 MHz

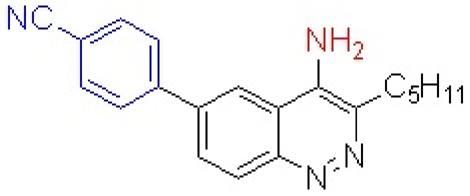

6

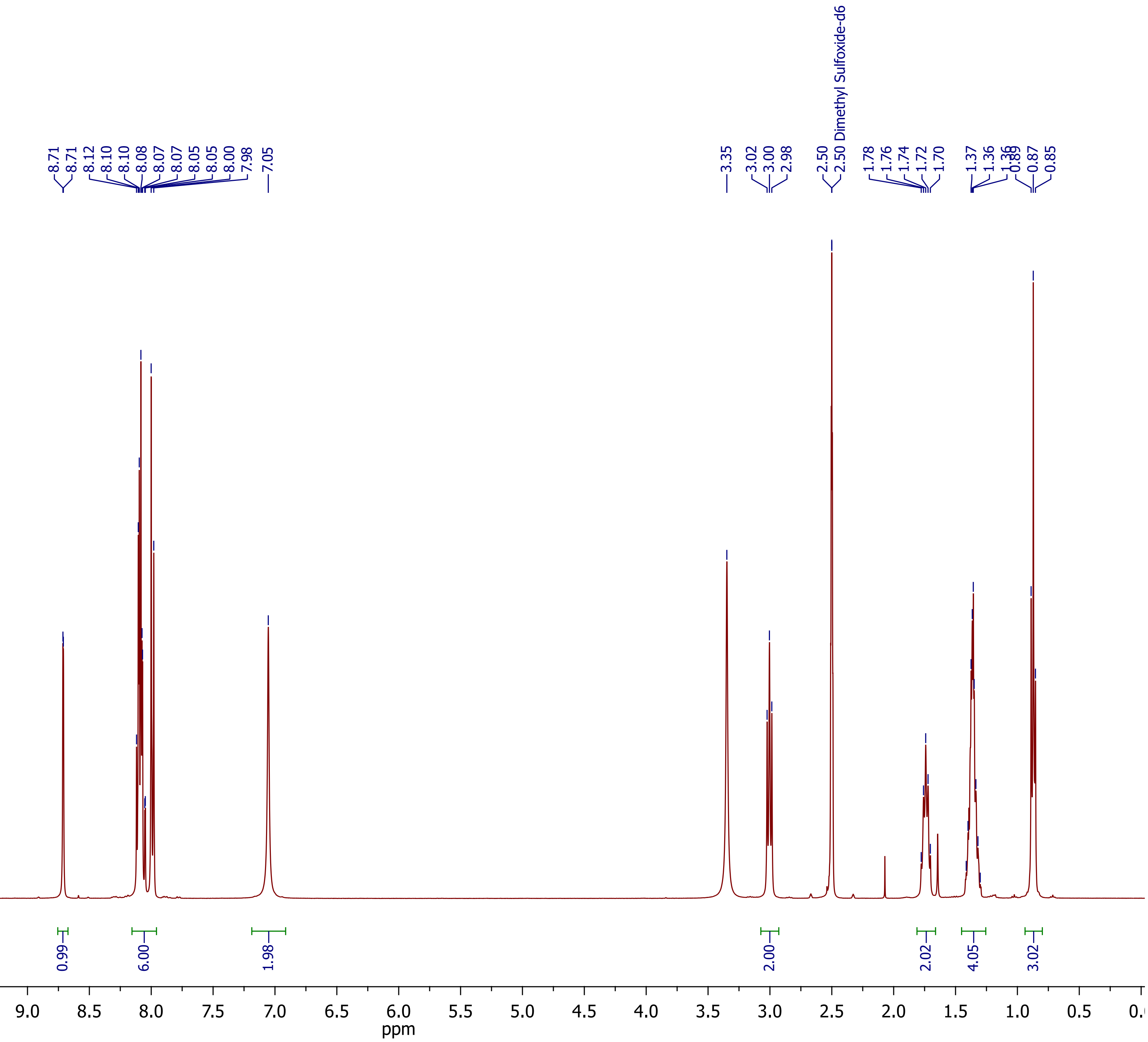

^{13}C NMR, DMSO-d_6, 101 MHz

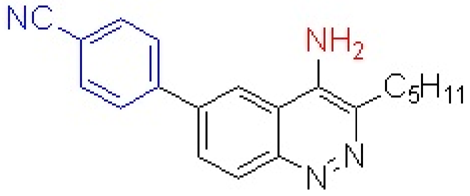

6

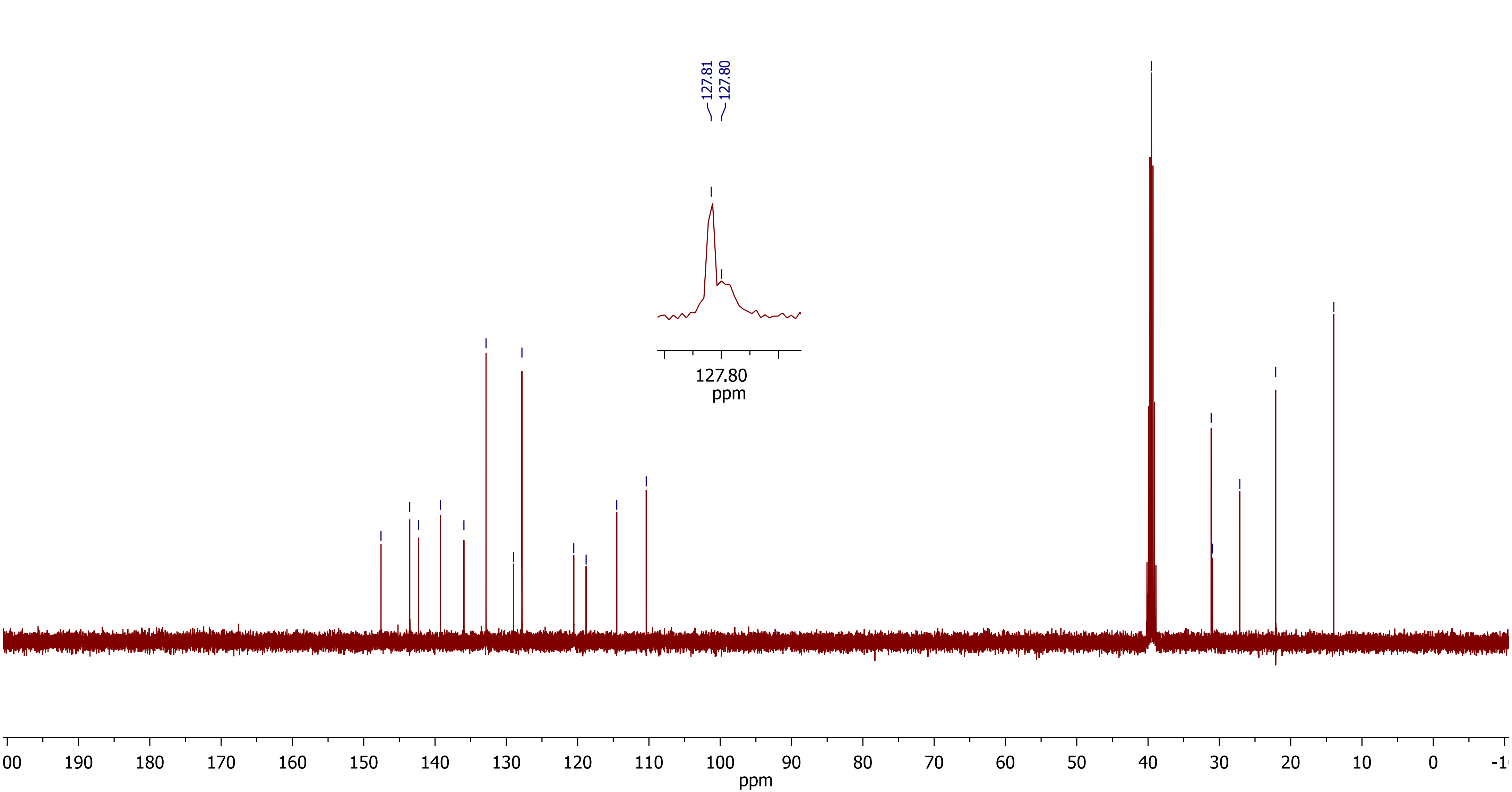

DEPT NMR, DMSO-d_6, 101 MHz

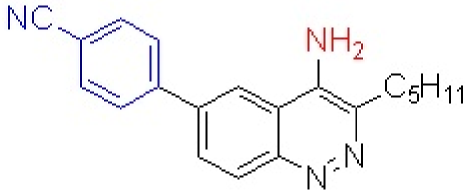

6

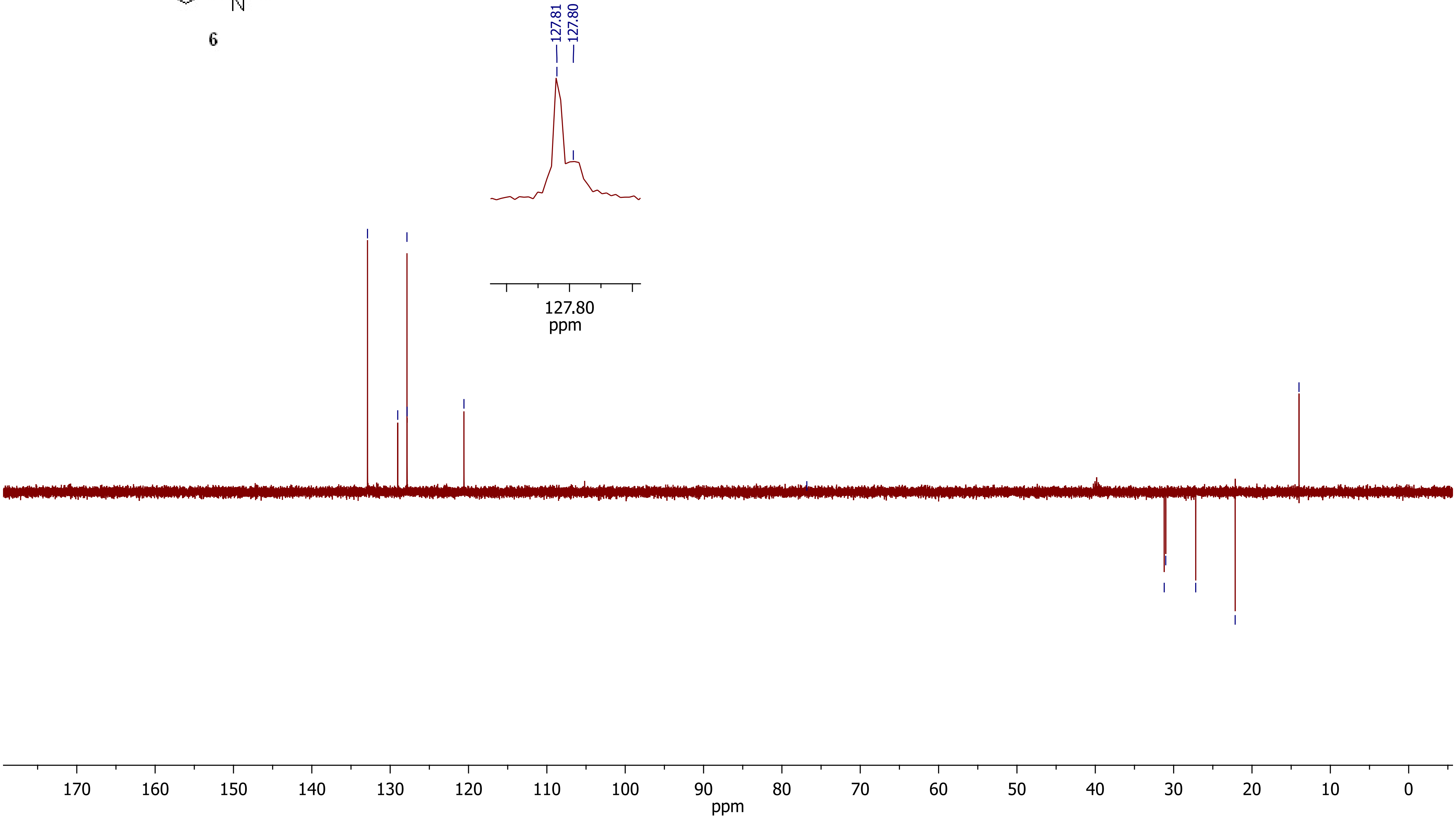

Mass Spectrum Report

Analysis Info

Analysis Name	D:\Data\2020\february\20\E6.d	Acquisition Date	20.02.2020 16:33:49
Method	tune_low.m	Operator	Bruker Customer
Sample Name	E6	Instrument / Ser#	micrOTOF 10223
Comment	MeOH		

Acquisition Parameter

Source Type	ESI	Ion Polarity	Positive	Set Nebulizer	0.4 Bar
Focus	Not active			Set Dry Heater	180 °C
Scan Begin	50 m/z	Set Capillary	4500 V	Set Dry Gas	4.0 l/min
Scan End	3000 m/z	Set End Plate Offset	-500 V	Set Divert Valve	Source

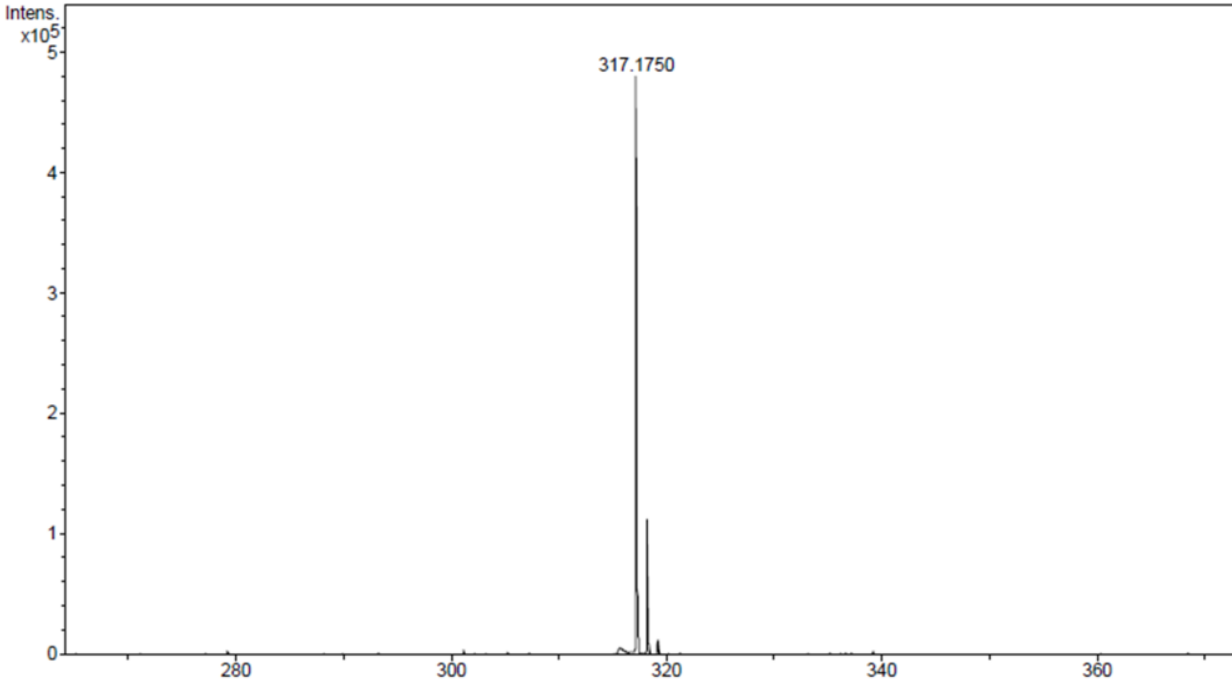

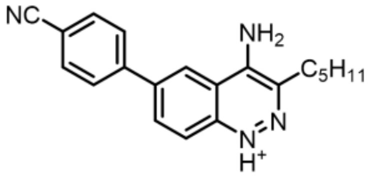

Chemical Formula: $C_{20}H_{21}N_4^+$
Exact Mass: 317,1761

Bruker Compass DataAnalysis 4.0 printed: 20.02.2020 16:35:53 Page 1 of 1

^{1}H NMR, acetone-d_6, 400 MHz

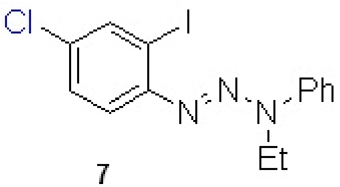

7

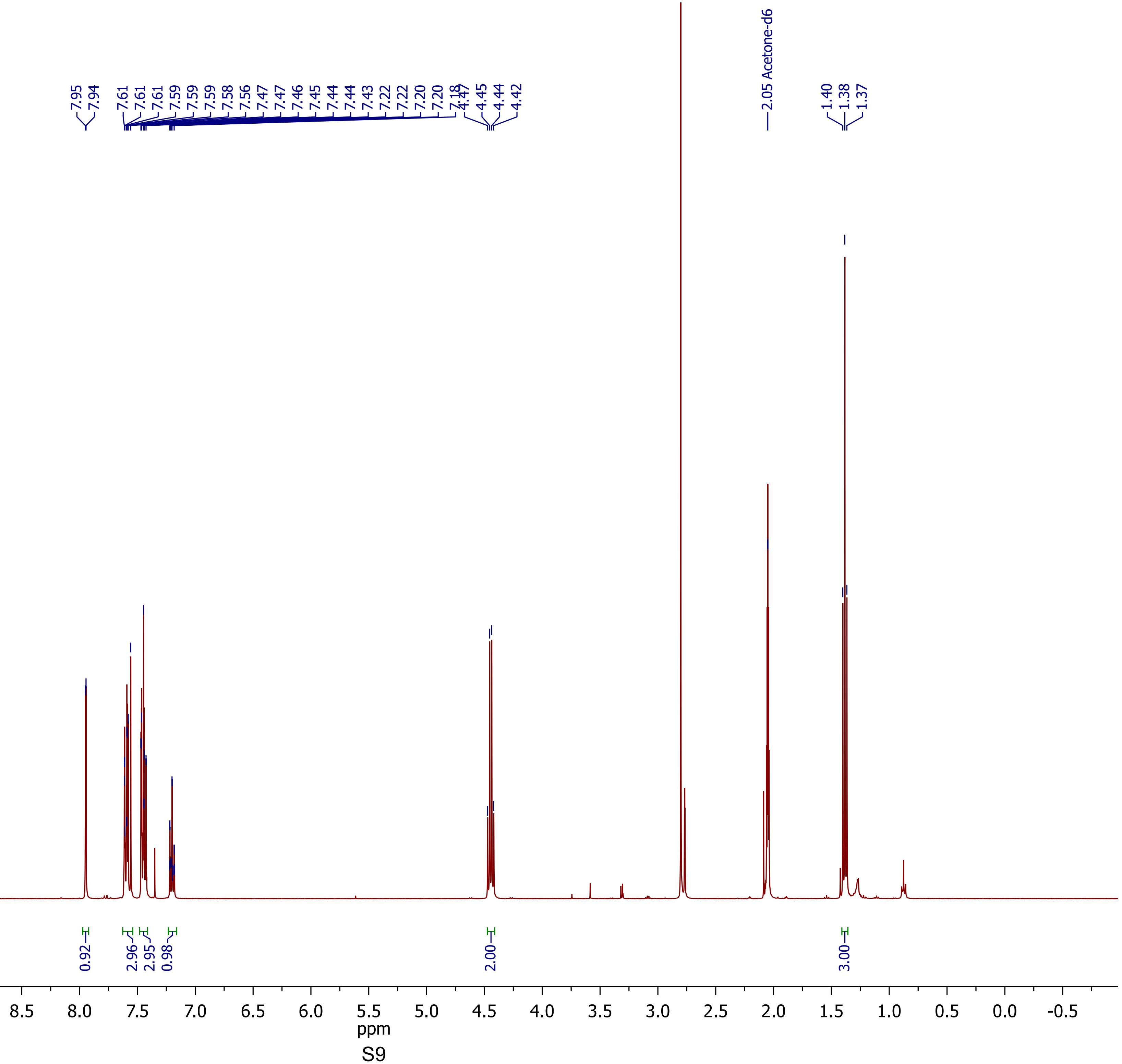

^{13}C NMR, acetone-d_6, 101 MHz

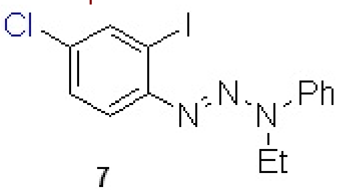

7

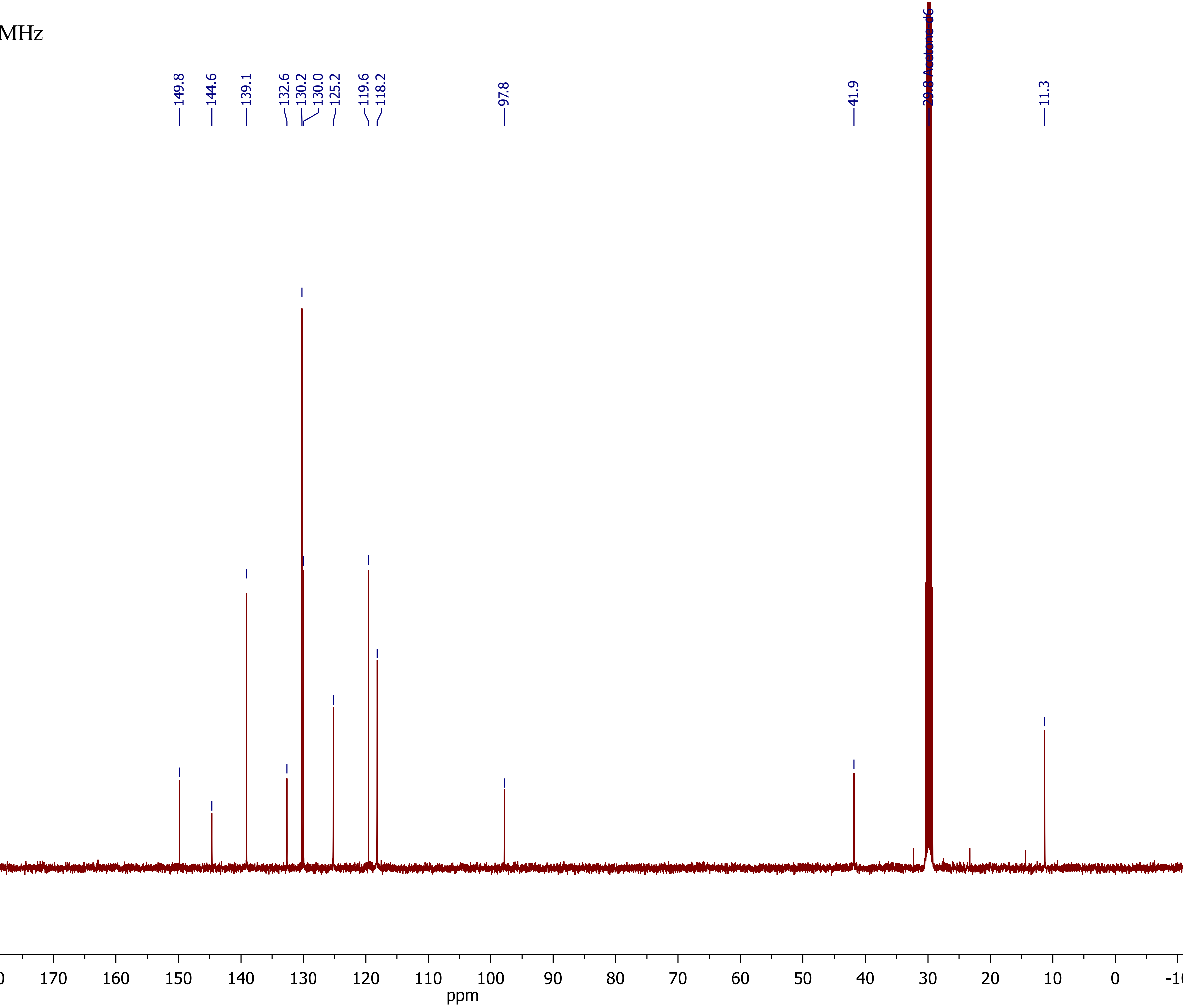

DEPT NMR, acetone-d_6, 101 MHz

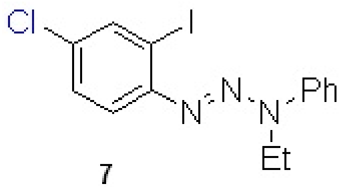

7

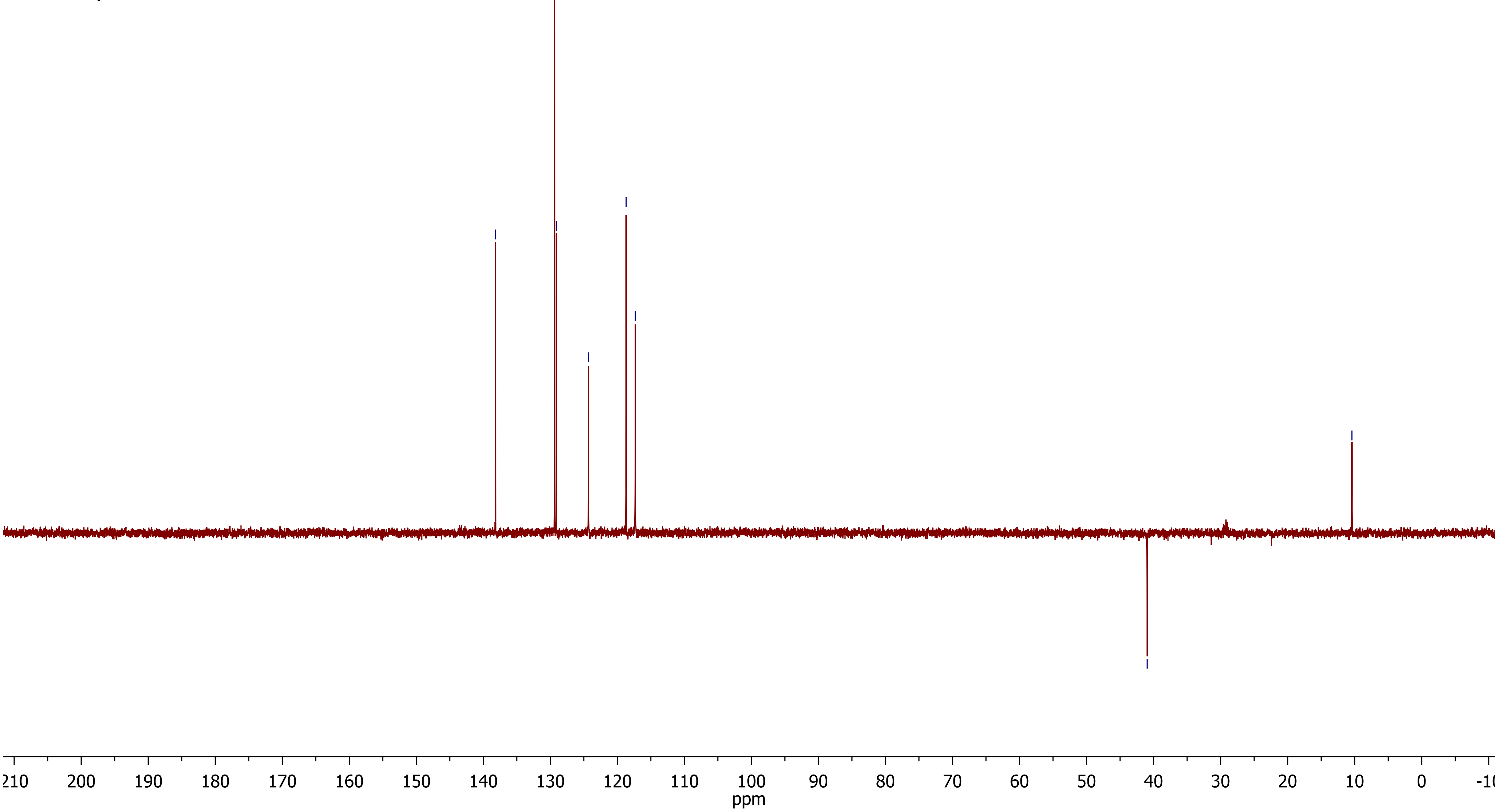

^{1}H NMR, acetone-d_6, 400 MHz

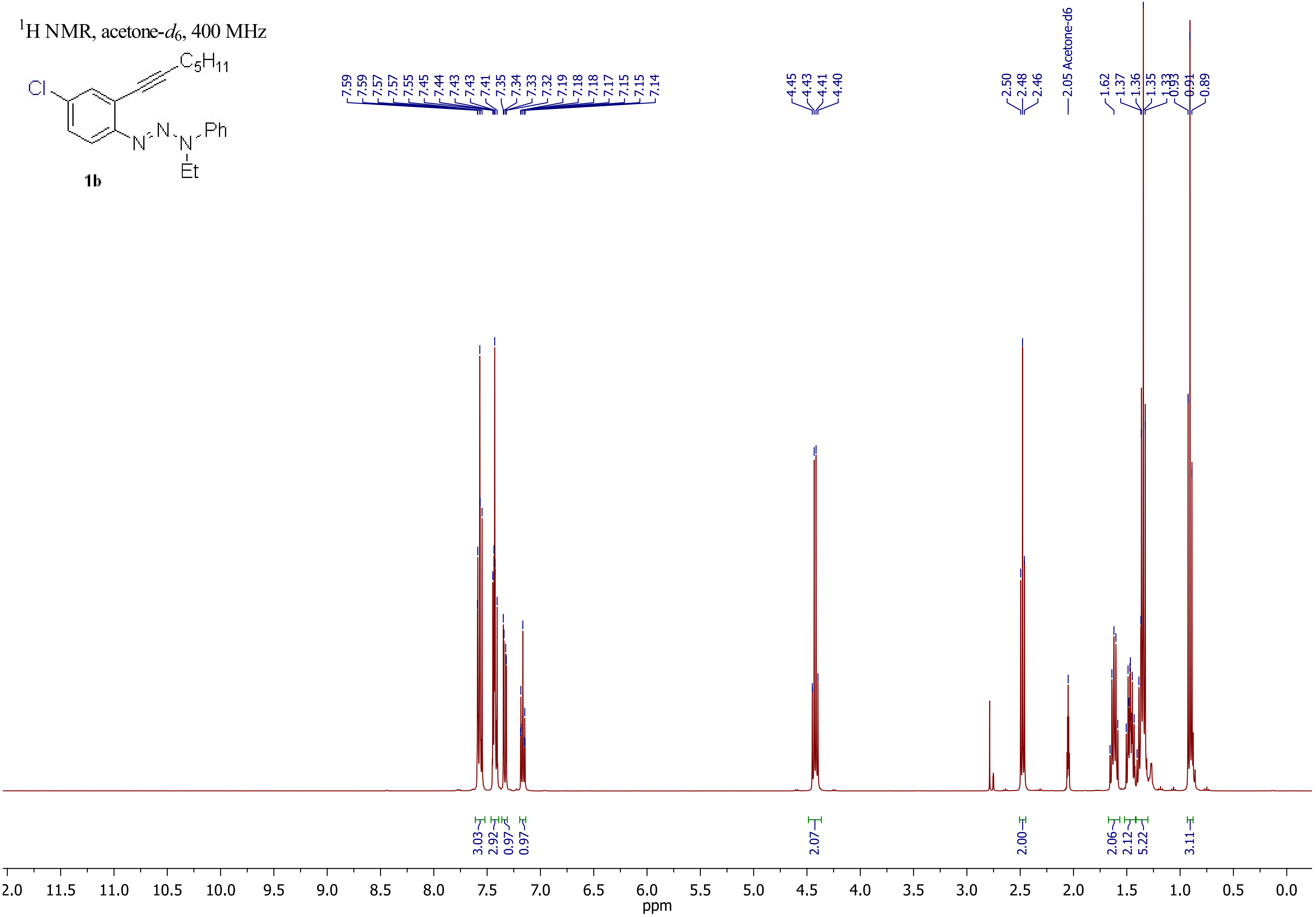

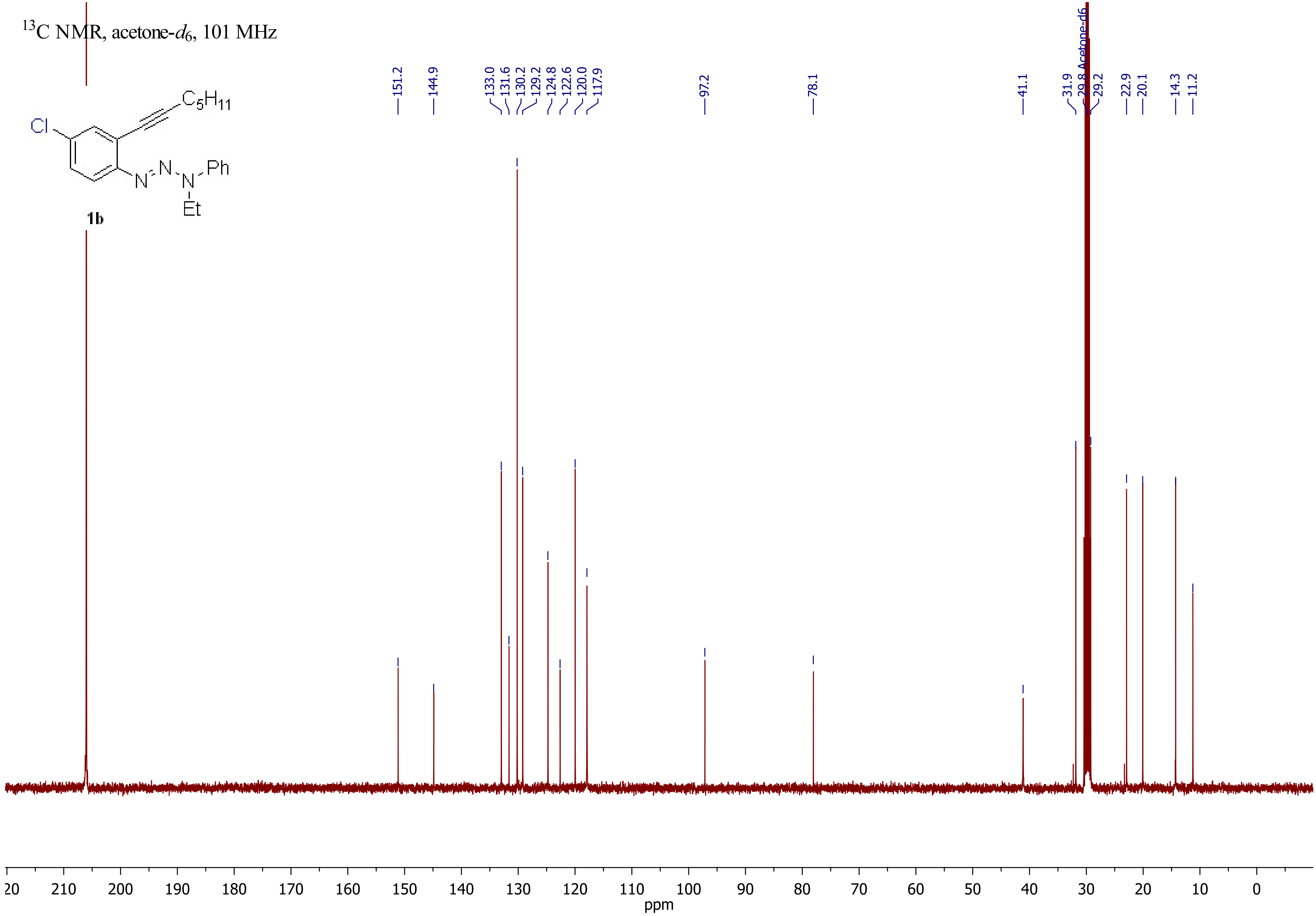

^{13}C NMR, acetone-d_6, 101 MHz
1b
151.2
144.9
133.0
131.6
130.2
129.2
124.8
122.6
120.0
117.9
97.2
78.1
41.1
31.9
29.8 Acetone-d6
29.2
22.9
20.1
14.3
11.2
ppm

DEPT NMR, acetone-d_6, 101 MHz

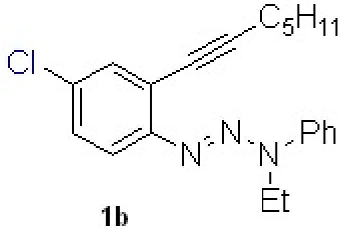

1b

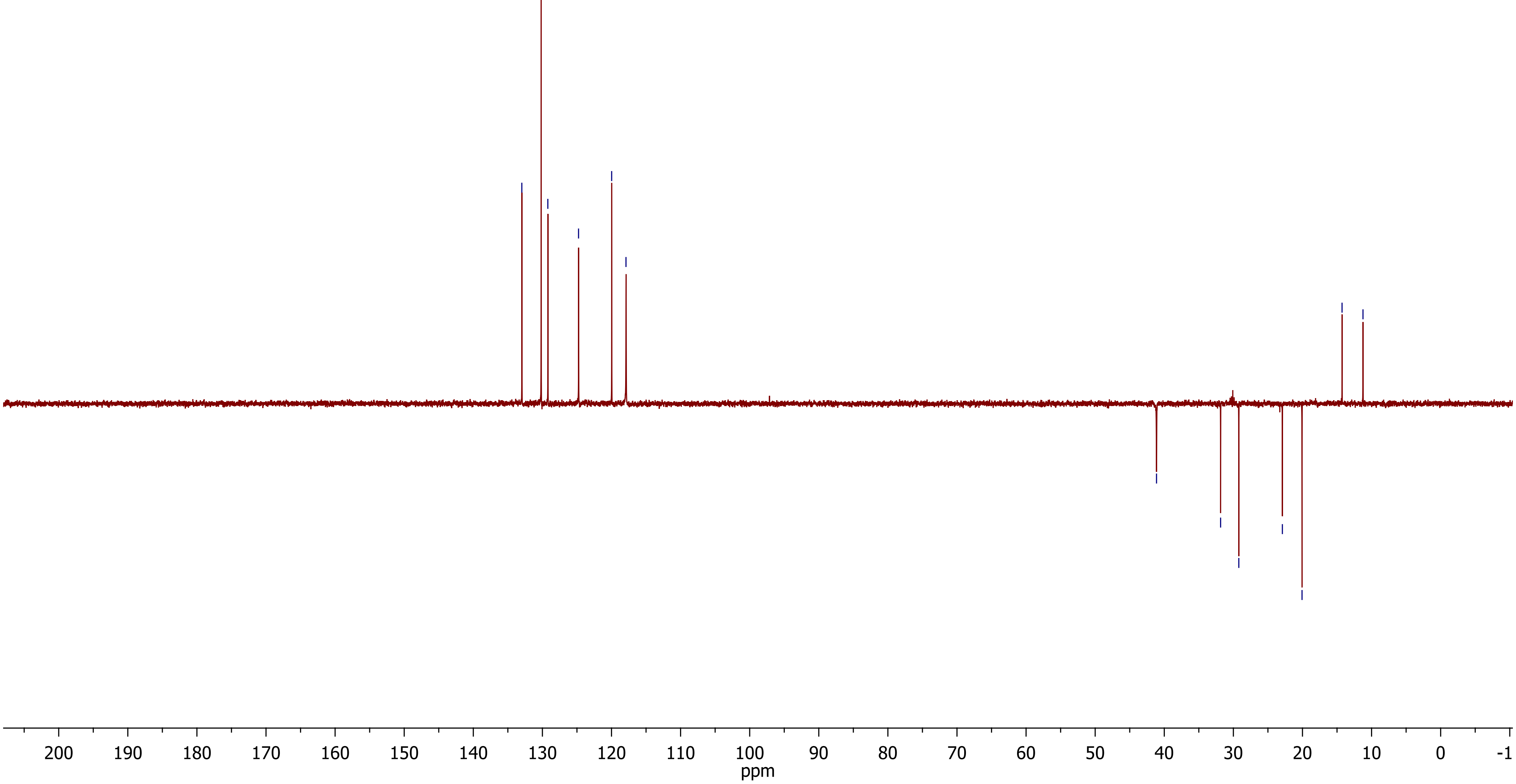

^{1}H NMR, acetone-d_6, 400 MHz

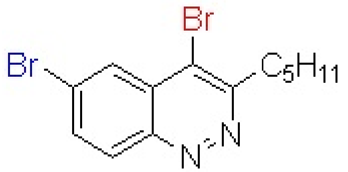

8a

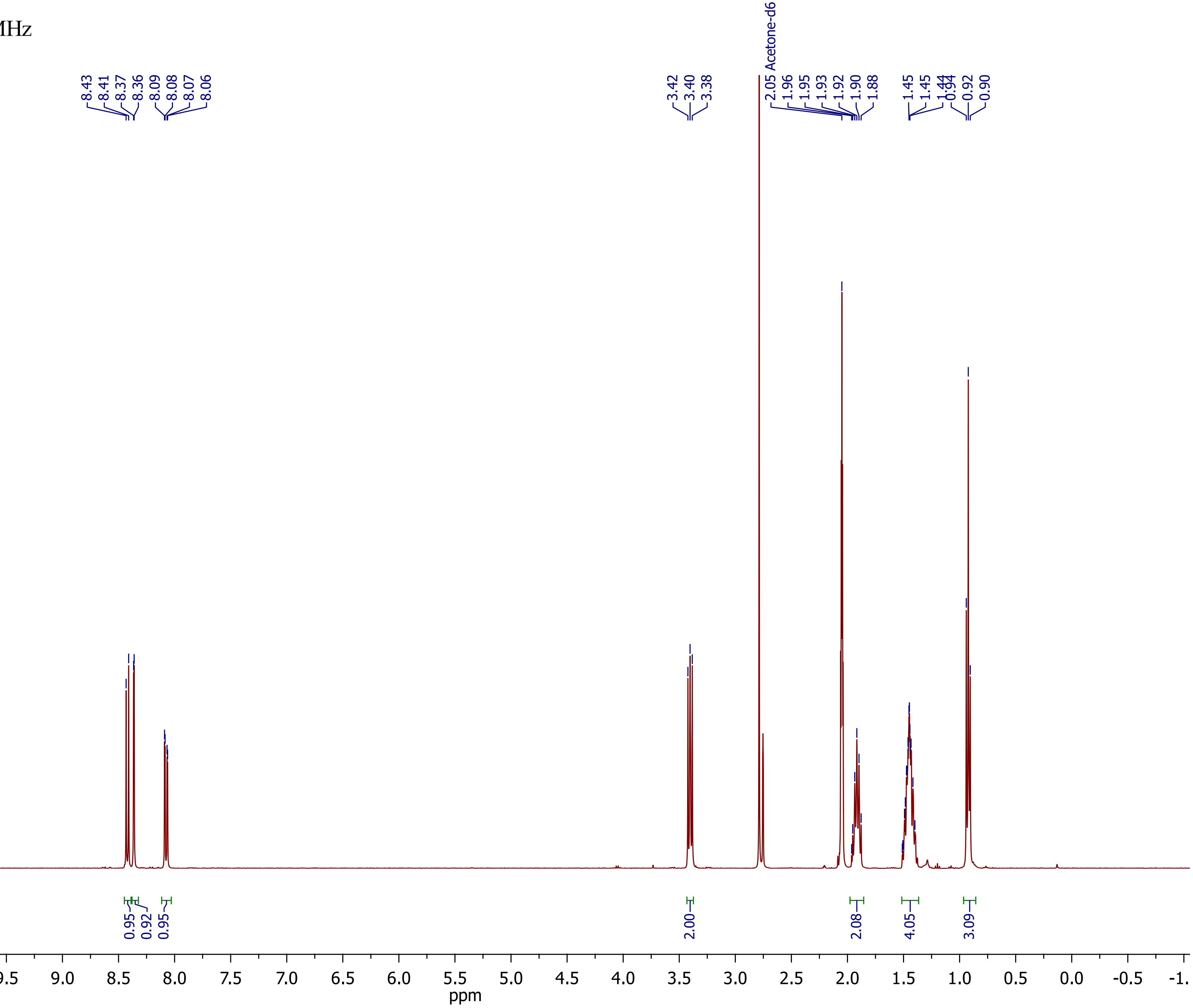

^{1}H NMR, acetone-d_6, 400 MHz

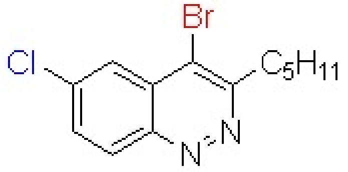

8b

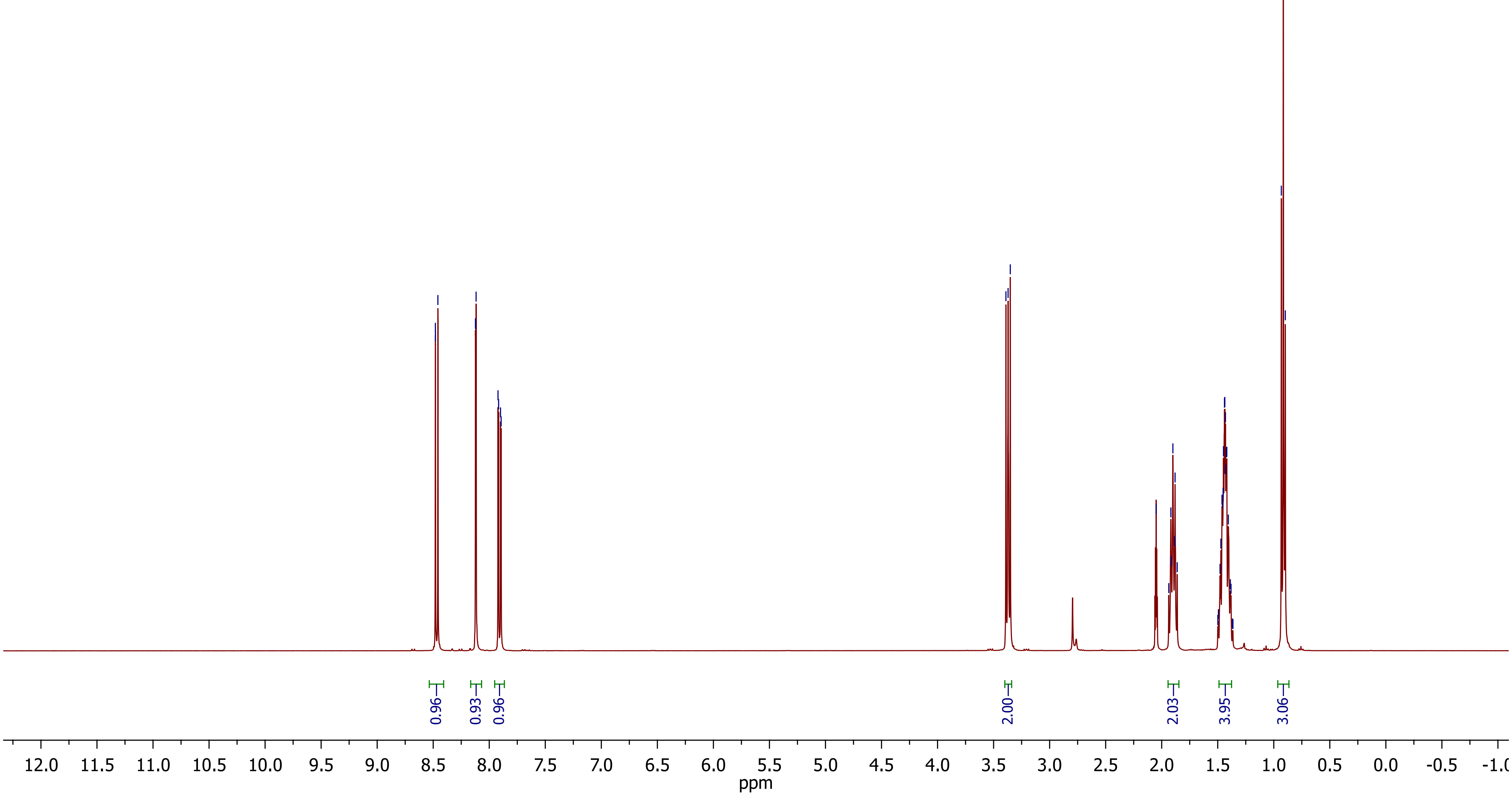

^{13}C NMR, acetone-d_6, 101 MHz

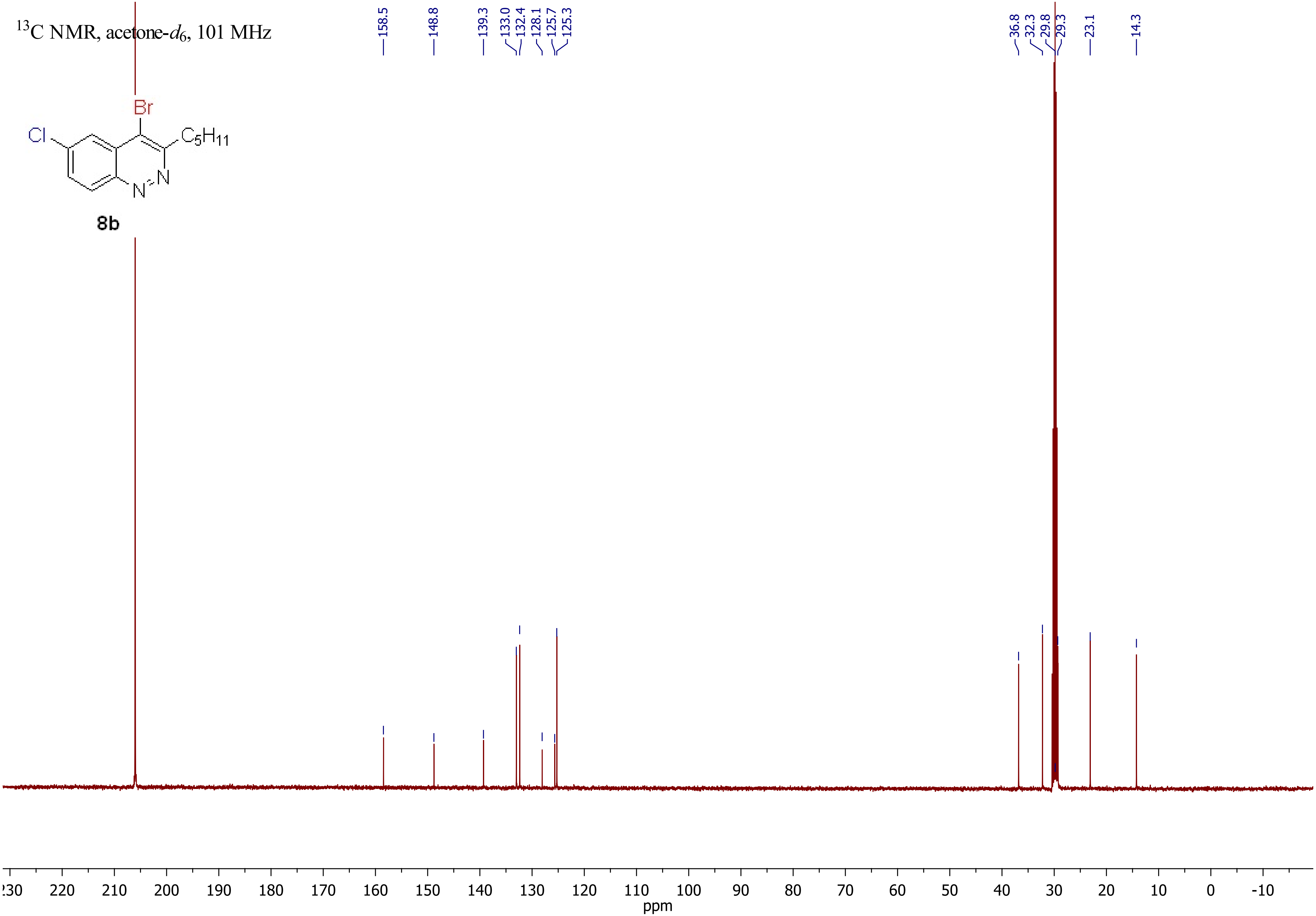

DEPT NMR, acetone-d_6, 101 MHz

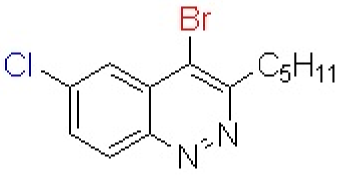

8b

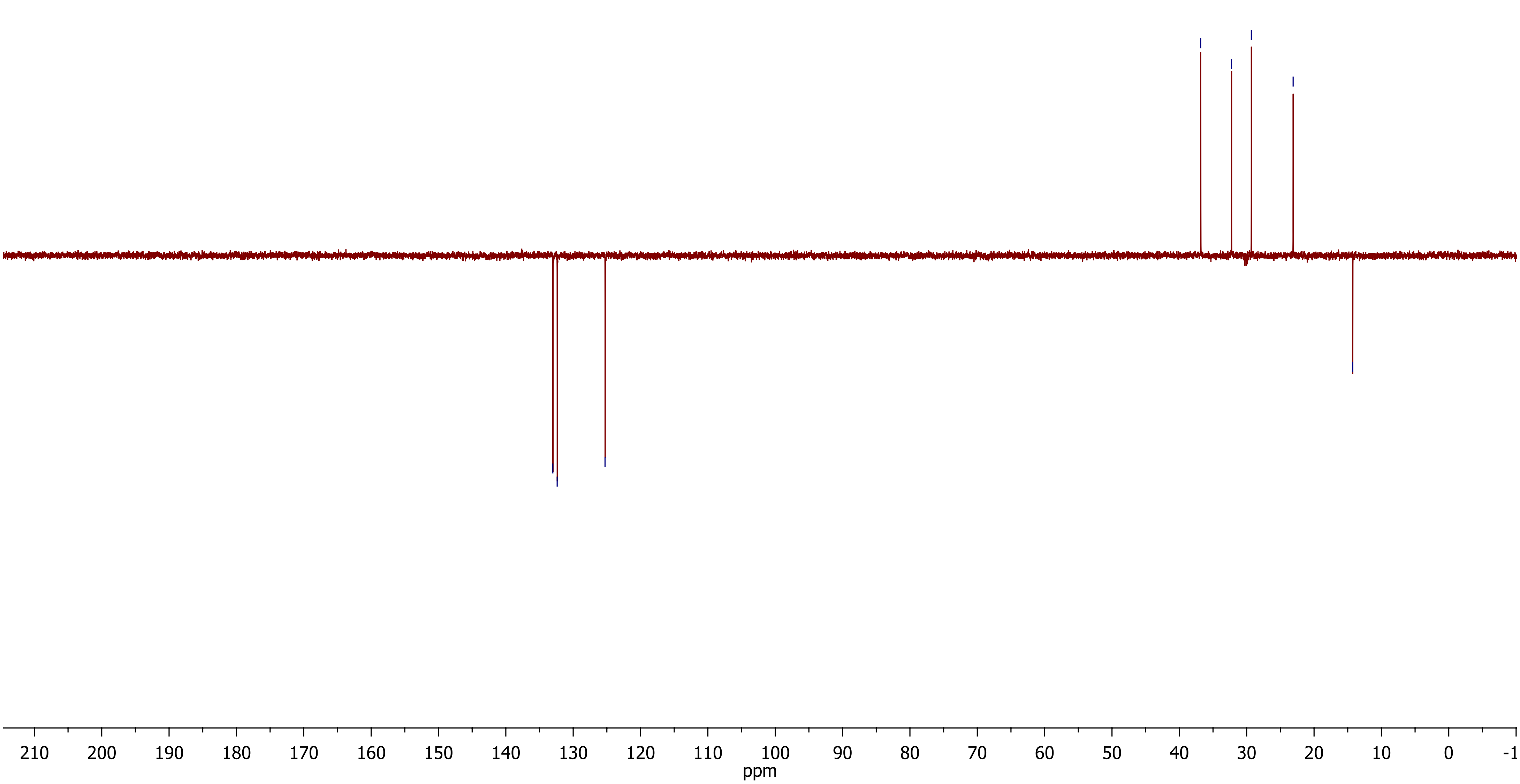

^{1}H NMR, acetone-d_6, 400 MHz

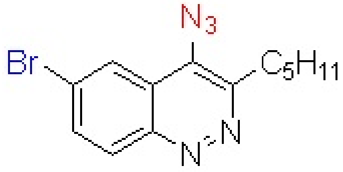

9a

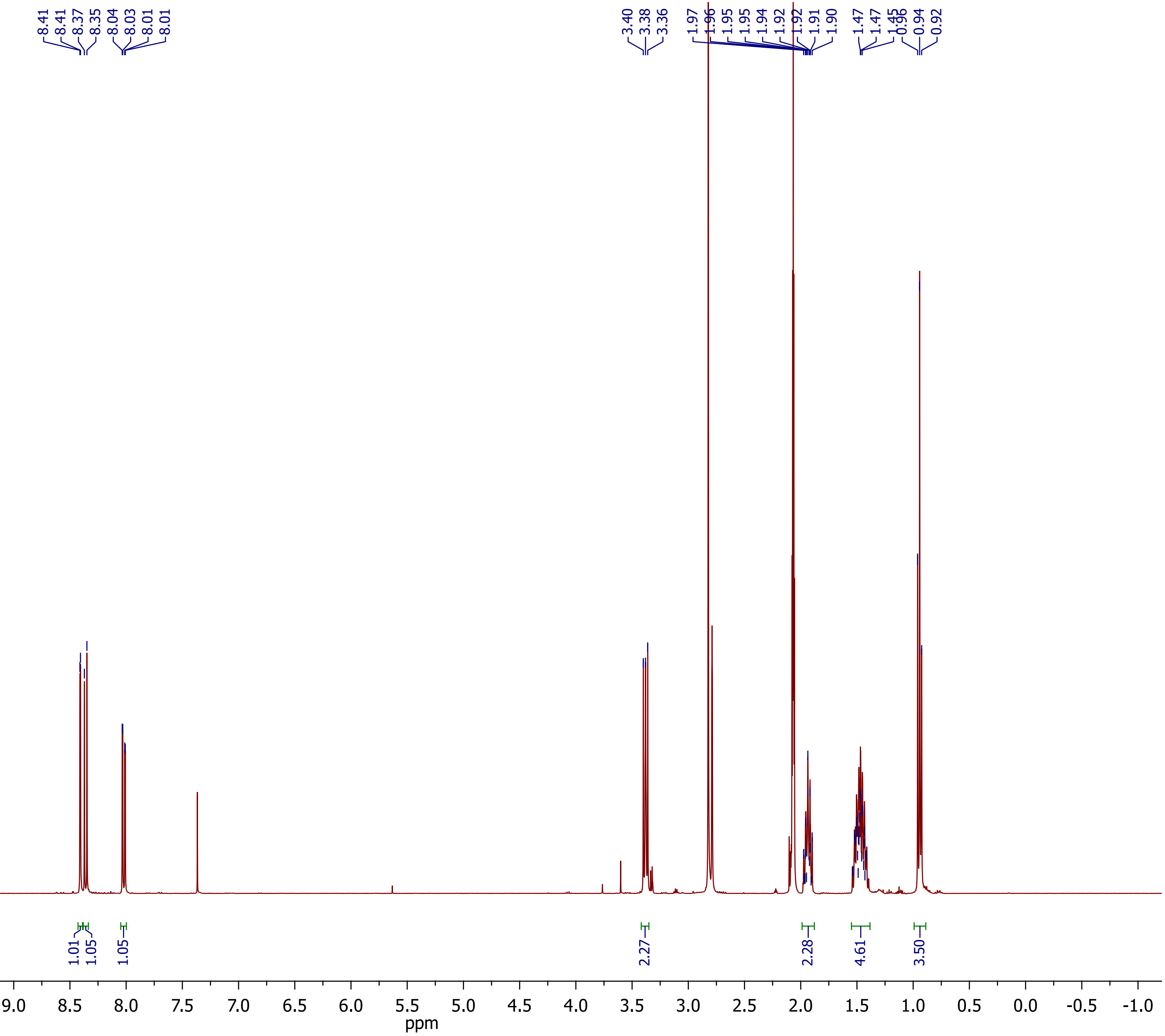

^{1}H NMR, acetone-d_6, 400 MHz

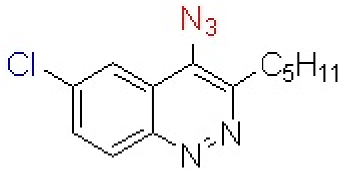

9b

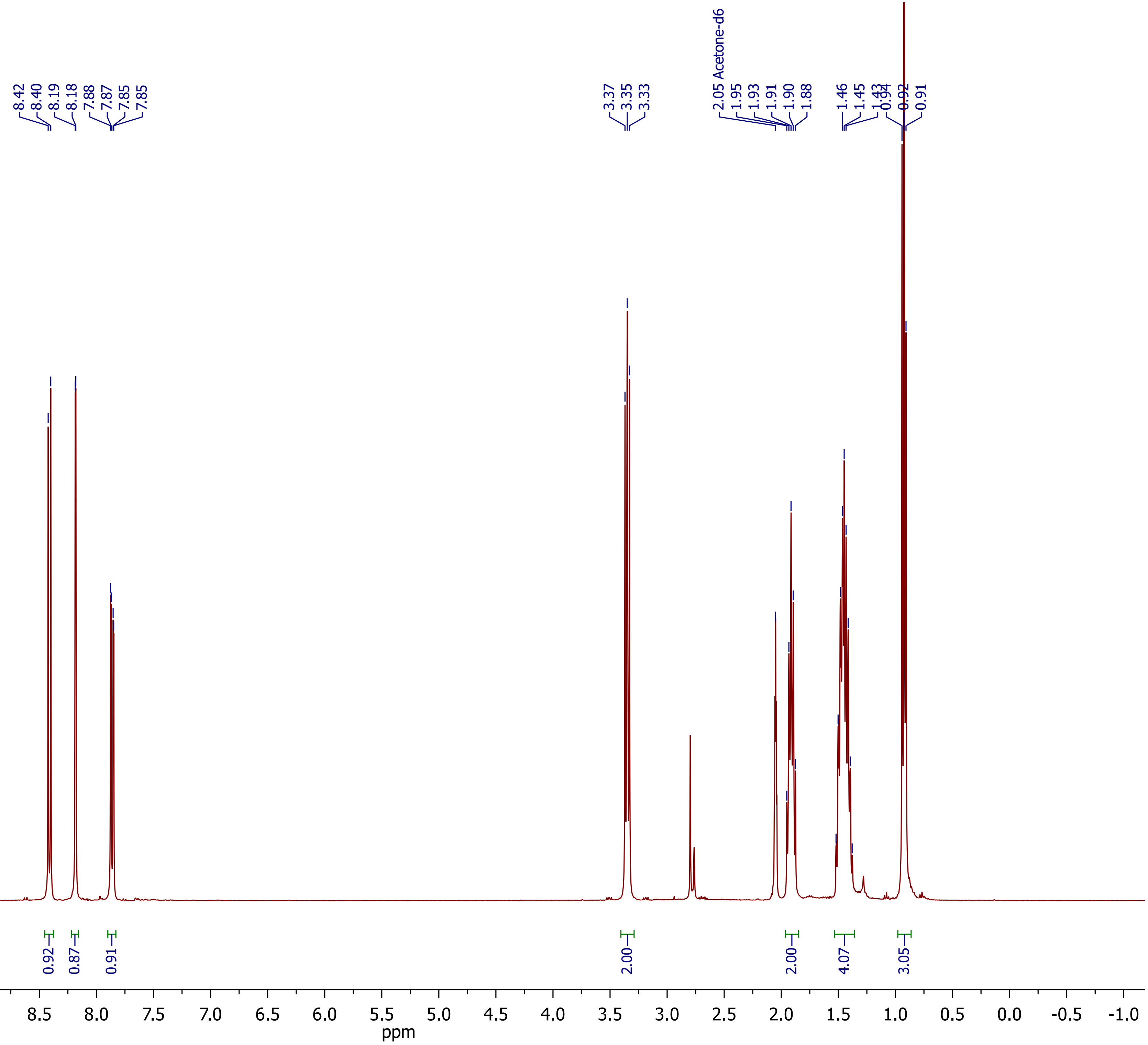

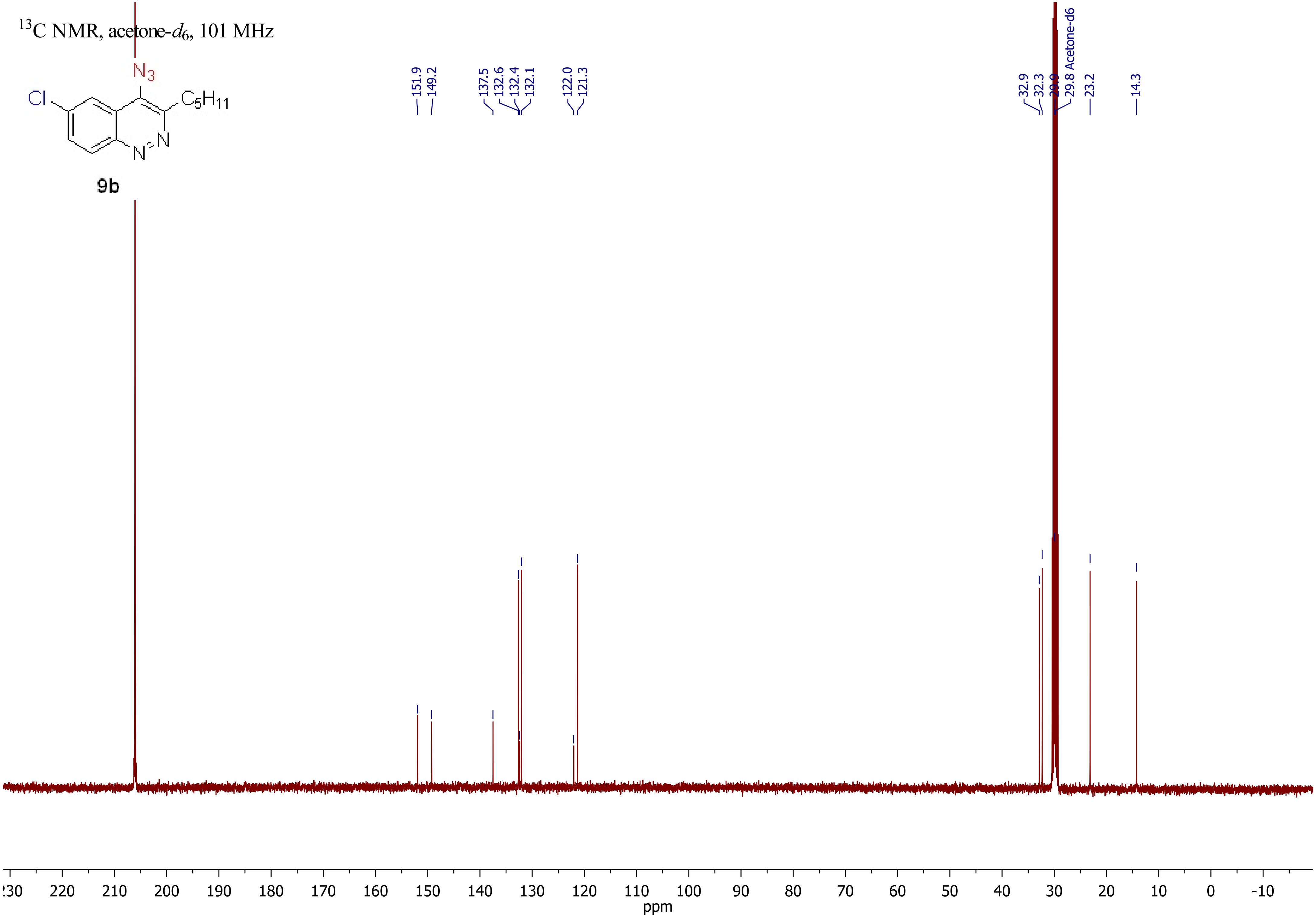

13C NMR, acetone-d6, 101 MHz
N3
Cl
C5H11
N
N
9b
151.9
149.2
137.5
132.6
132.4
132.1
122.0
121.3
32.9
32.3
29.9
29.8 Acetone-d6
23.2
14.3
230
220
210
200
190
180
170
160
150
140
130
120
110
100
90
80
70
60
50
40
30
20
10
0
-10
ppm

DEPT NMR, acetone-d_6, 101 MHz

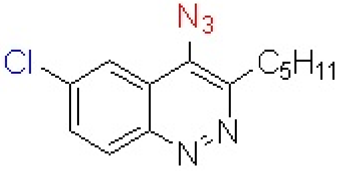

9b

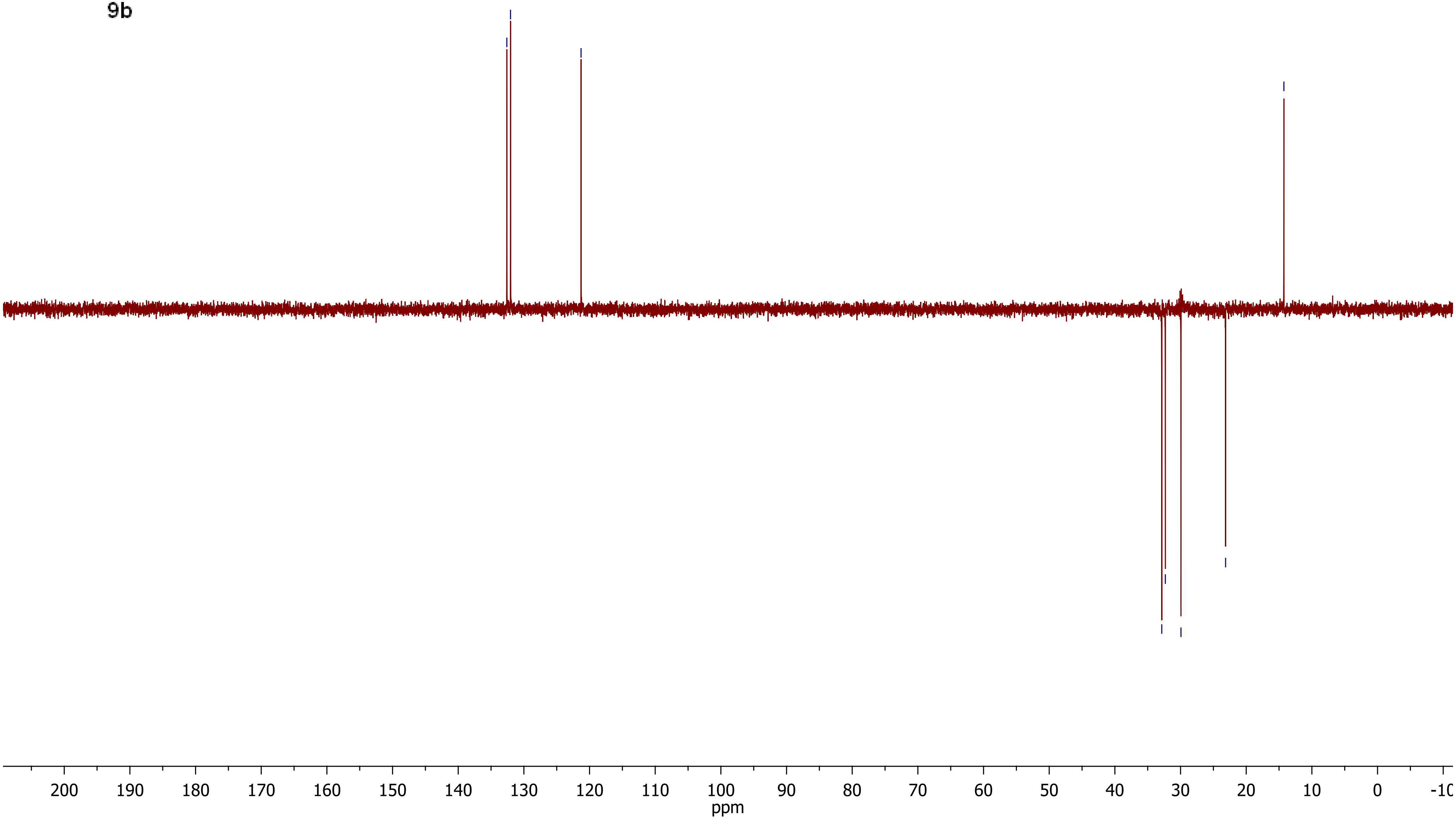

^{1}H NMR, DMSO-d_6, 400 MHz

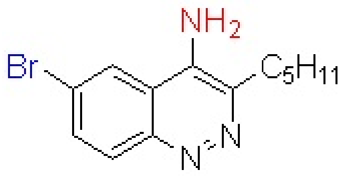

10a

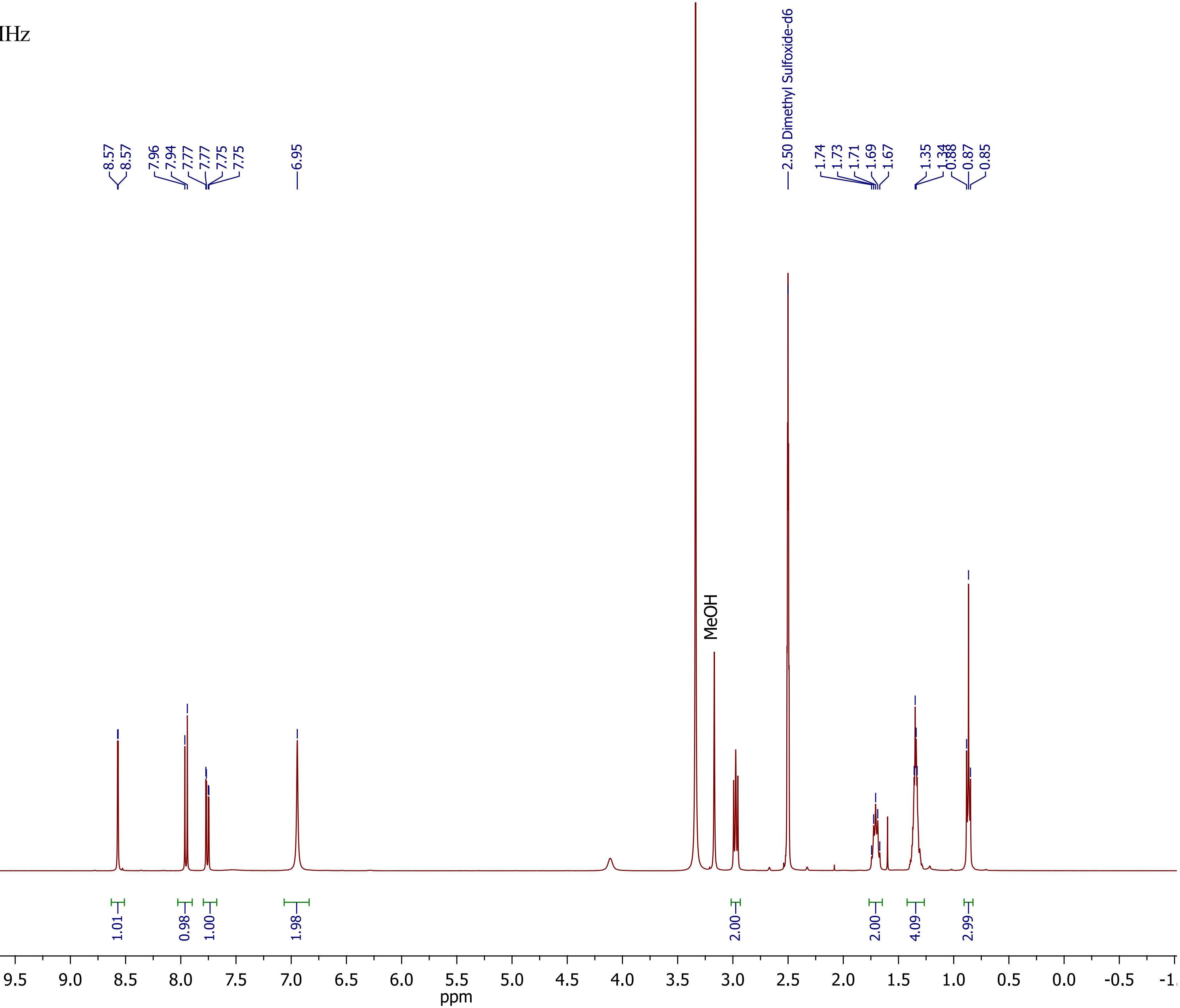

^{13}C NMR, DMSO-d_6, 101 MHz

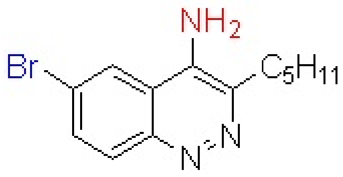

10a

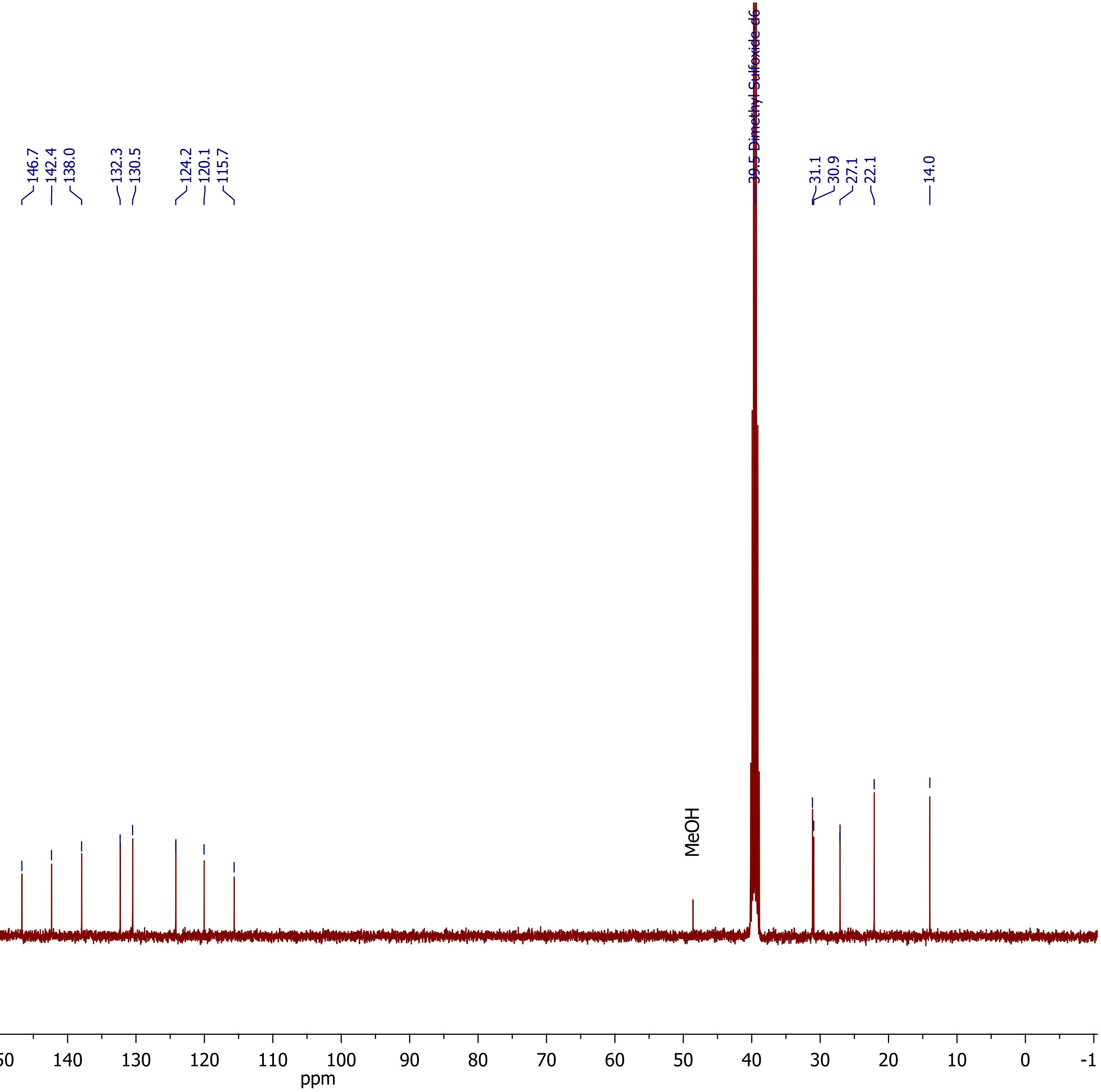

DEPT NMR, DMSO-d_6, 101 MHz

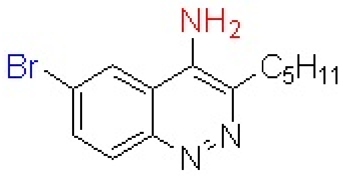

10a

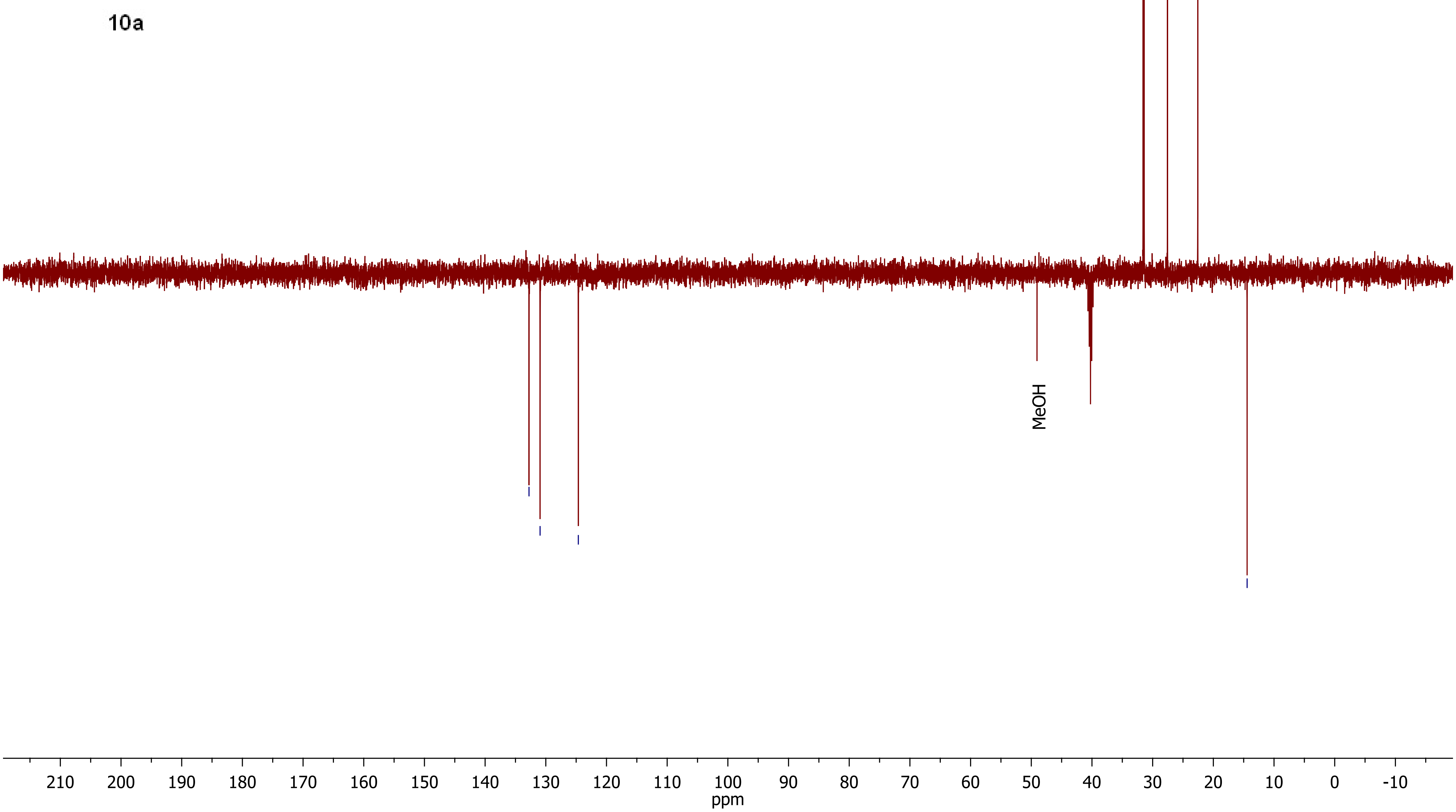

^{1}H NMR, DMSO-d_6, 400 MHz

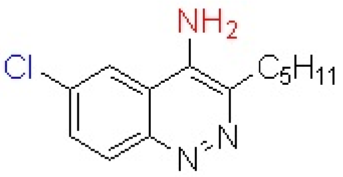

10b

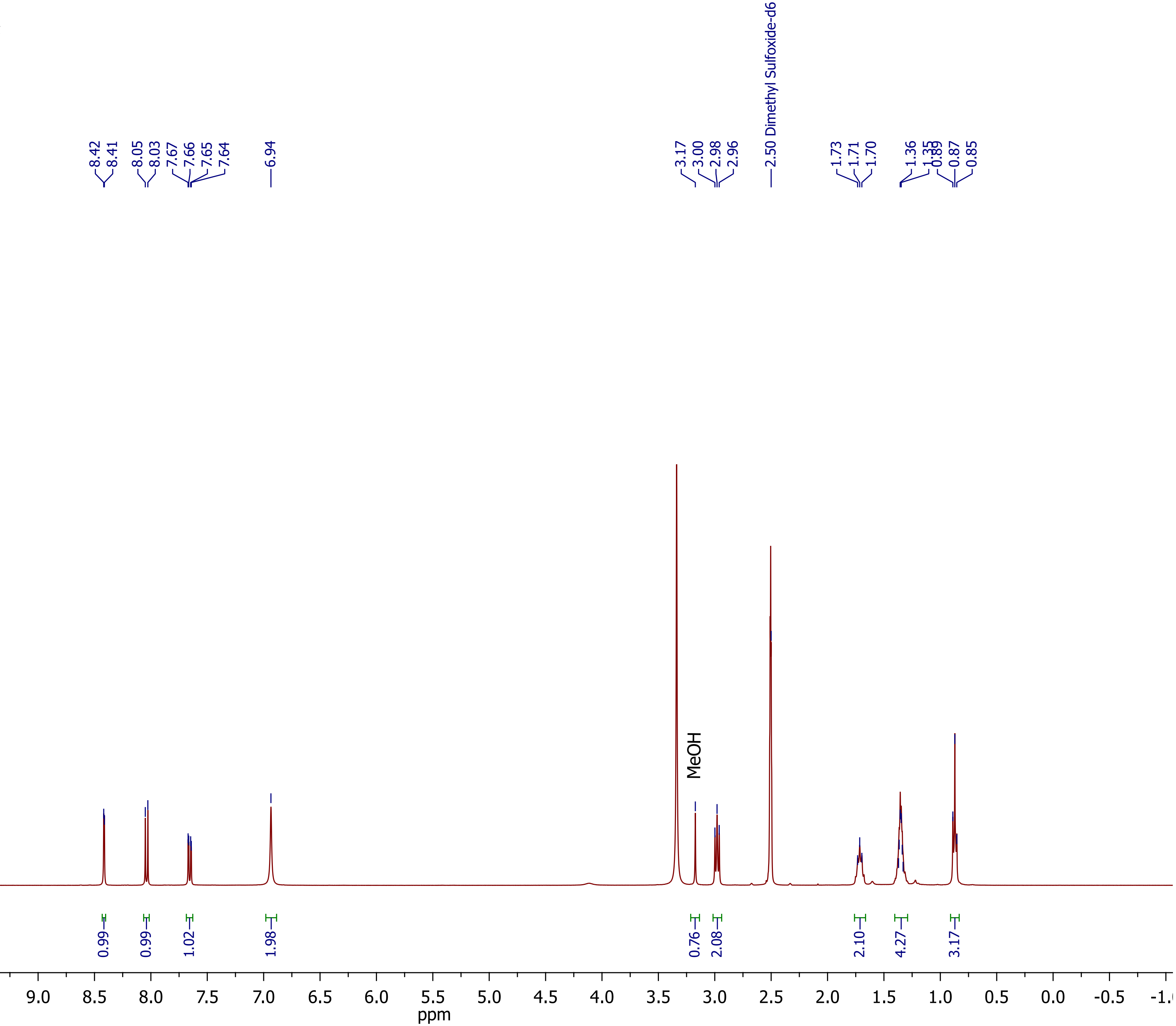

^{13}C NMR, DMSO-d_6, 101 MHz

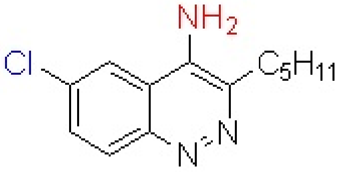

10b

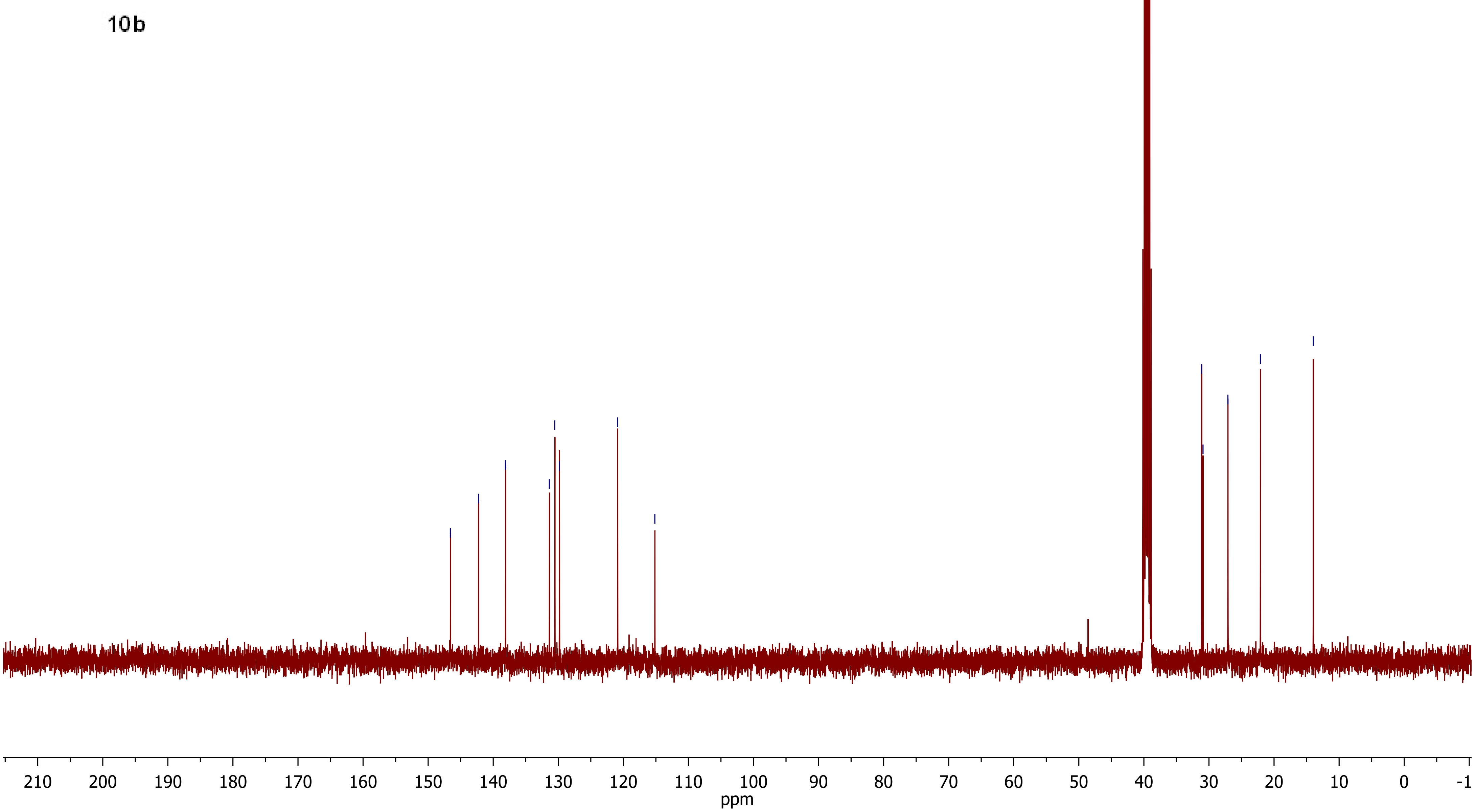

DEPT NMR, DMSO-d_6, 101 MHz

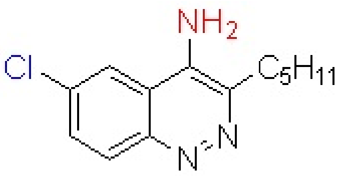

10b

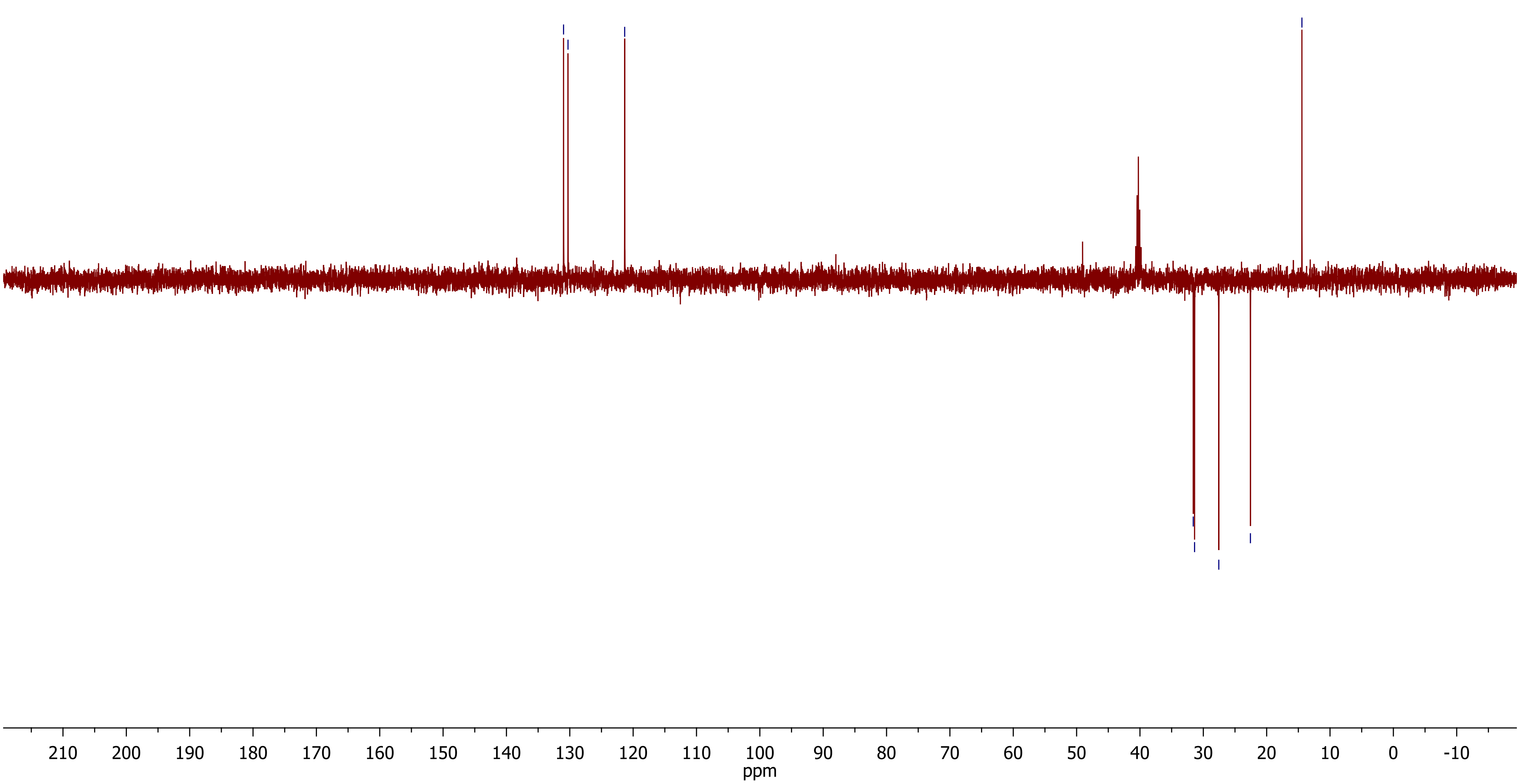

Details of HPLC Measurements and Copies of HPLC Chromatograms

Table S1. Calibration equation and R^2 for amine **6** and azide **5**

Compound	Calibration equation	R^2
Amine **6**	Y = aX, a = 2.271414e-005	0.9992
Azide **5**	Y = aX, a = 4.93494e-005	0.9897

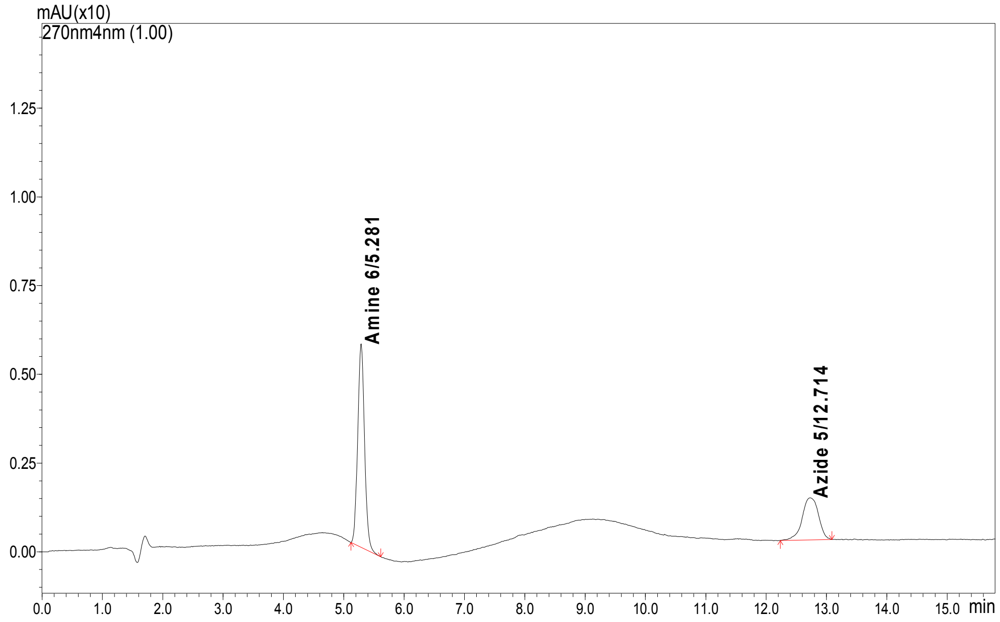

Fig. S1. A typical chromatogram of a standard solution of amine **6** and azide **5** (1μg/mL) by RP HPLC with photodiode array detection (270 nm). HPLC conditions: eluent composition - water with 0.1% HCOOH (solvent A) and acetonitrile with 0.1% HCOOH (solvent B); gradient elution mode 30–70% of solvent B for 10 min; flow rate 0.3 ml/min, sample injected volume 2 μL; UV: 270 nm.

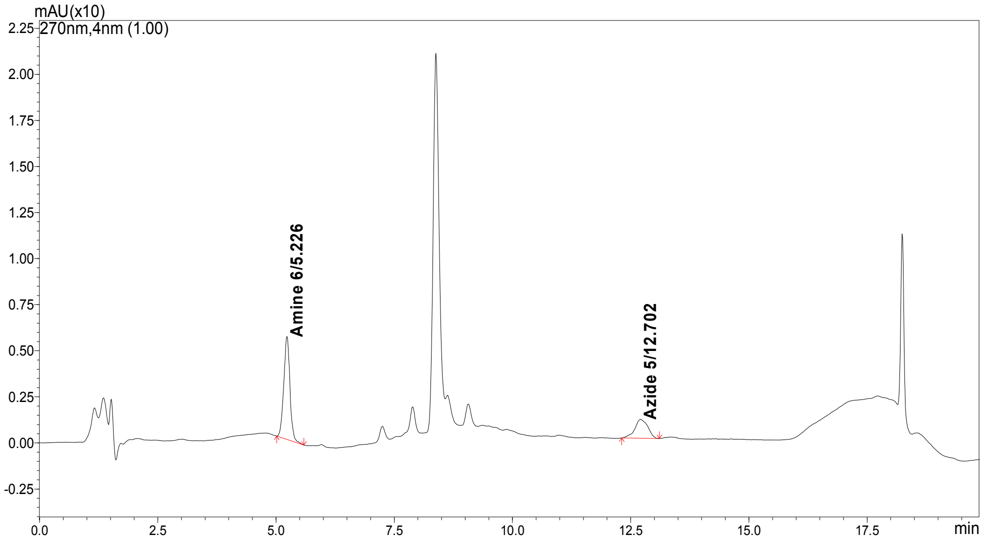

Fig. S2. Chromatogram of HepG2 cell lysate treated with azide **5** by RP HPLC with photodiode array detection (270 nm). HPLC conditions the same as for Fig. S1.

Table S2. Concentrations of azide **5** and amine **6** in the samples of cells lysate after the incubation of cells with azide **5**.

	Retention time, min	Concetntration, μg/ml	Concetntration, μM	Amine **6** to azide **5** ration
Amine **6**	5.23	1.14±0.02	3.60±0.06	1.23
Azide **5**	12.7	1.00±0.03	2.92±0.09	

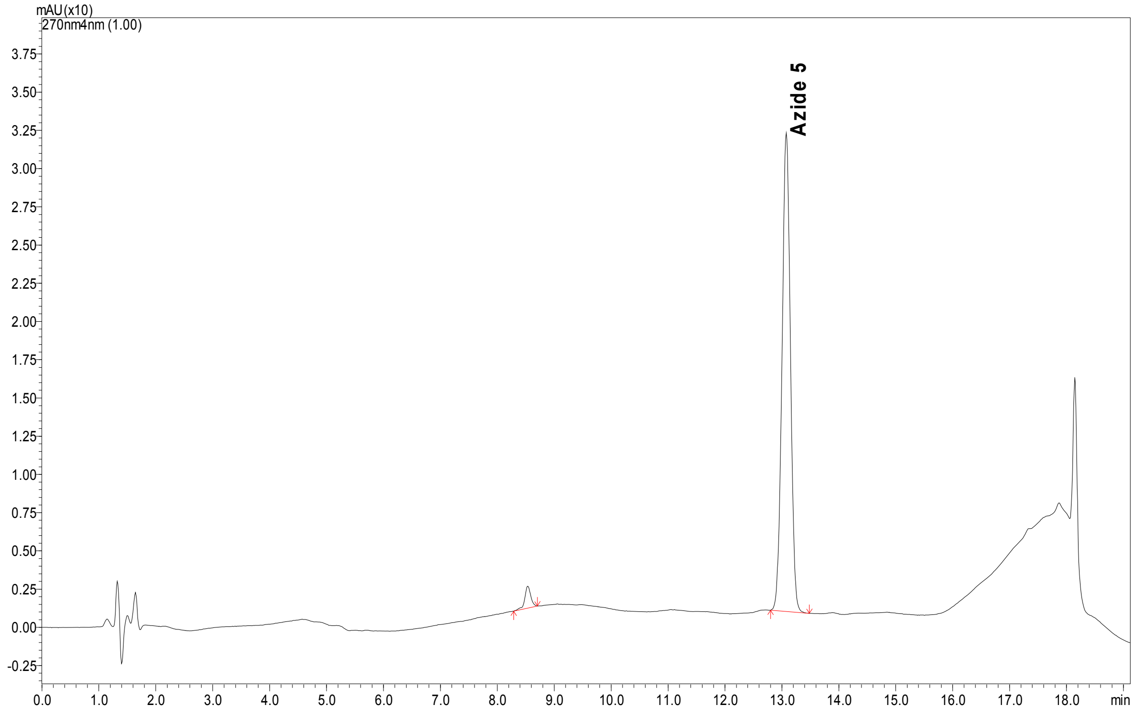

Fig. S3. A typical chromatogram of a standard solution of azide **5** by RP HPLC with photodiode array detection (270 nm). HPLC conditions the same as for Fig. S1.

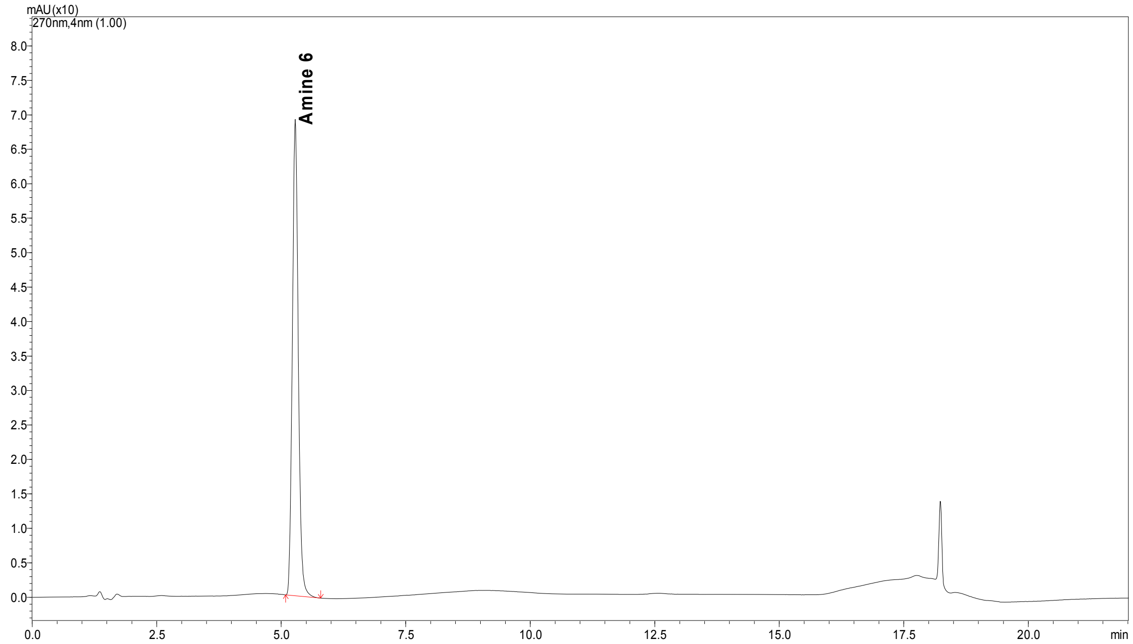

Fig. S4. A typical chromatogram of a standard solution of amine **6** by RP HPLC with photodiode array detection (270 nm). HPLC conditions the same as for Fig. S1.

Details of QY Fluorescence Measurements

Table S3. Preparation of stock solution and working solutions of azide **5** and amine **6** for the measurements of absolute quantum yields (QY).

Compound	Mass, mg	Mw, g/mol	Volume of THF (μL) for the preparation of stock solution ($c = 1\times10^{-3}$ mol/L)	Concentration of the working solution, mol/L / Solvent	Volume of the stock solution for the preparation of working solutions (10 mL)	Volume of solvents (THF, *i*-PrOH, H_2O) for the preparation of working solutions (10 mL)
Azide **5**	1.00	342.4	2920	5×10^{-6} / THF	50 μL	9950 μL for each sample
				5×10^{-6} / H_2O	50 μL	
Amine **6**	0.80	316.2	2530	1.4×10^{-6} / THF	140 μL	
				1.4×10^{-6} / MeCN	140 μL	
				1.4×10^{-6} / *i*-PrOH	140 μL	
				1.4×10^{-6} / *i*-H_2O	140 μL	

Experimental details for the synthesis of amines 7a,b.

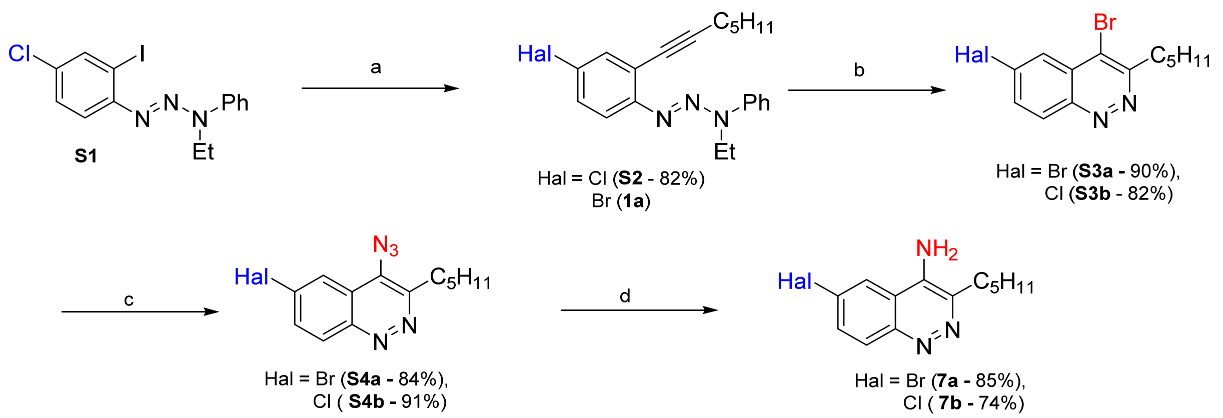

Reagents and conditions: *a* – hept-1-yne (1.5 equiv), $Pd(PPh_3)_4$ (2.5 mol%), CuI (10 mol%), KF (5 equiv), DMF, 40 °C; *b* – HBr (20 eqiuv), acetone, 0.1 M, r.t., 10 min; c – NaN_3(5 equiv), 50 °C, 24h; *d* – $NaBH_4$(1.5 eqiuv), MeOH, r.t., 1 h.

Scheme S1. Synthesis of 6-bromo- and 6-chlorocinnoline-4-amines **10a,b**.

(*E*)-1-(4-chloro-2-iodophenyl)-3-ethyl-3-phenyltriaz-1-ene (S1): Triazene **S1** was synthesized in accordance with the know procedure described for (*E*)-1-(4-bromo-2-iodophenyl)-3-ethyl-3-phenyltriaz-1-ene triazene [1] from 4-chloro-2-iodoaniline (2.56 g, 10.1 mmol). Purification of the crude product by column chromatography using hexane / triethylamine (100 : 0.01) as the eluent gave 2.29 g of **S2** (59%). ^{1}H NMR (400 MHz, acetone-*d*$_6$) δ = 7.95 (d, *J*=2.3 Hz, 1H), 7.64 – 7.54 (m, 3H), 7.49 – 7.40 (m, 3H), 7.24 – 7.16 (m, 1H), 4.45 (q, *J*=7.0 Hz, 2H), 1.38 (t, *J*=7.0 Hz, 3H). ^{13}C NMR (101 MHz, acetone-*d*$_6$) δ = 149.8, 144.7, 139.1, 132.6, 130.2, 130.0, 125.2, 119.6, 118.2, 97.8, 41.8, 11.3. HRMS (ESI): *m*/*z* calcd for $C_{14}H_{13}ClN_3I$ +H$^+$: 385.9915 [*M*+H]$^+$; found: 385.9900.

(*E*)-1-(4-Chloro-2-(hept-1-yn-1-yl)phenyl)-3-ethyl-3-phenyltriaz-1-ene (S2): To a stirred degassed solution of triazene **S1** (2.28 g, 5.91 mmol, 1.00 equiv) in anhydrous DMF (30.0 mL, c = 0.2 M) were added KF (1.72 g, 29,6 mmol, 5.00 equiv), $Pd(PPh_3)_4$ (171 mg, 0.148 mmol, 2.5 mol%) and CuI (113 mg, 0.591 mmol, 10 mol%). The flask was degassed once again using a freeze-pump-thaw technique over three degassing cycles. After that, hept-1-yne (8.87 mmol, 853 mg, 0.765 mL, 1.50 equiv) was added with a syringe. The reaction mixture was stirred at 40 °C under an Ar atmosphere for 24 hours (TLC control). Then the reaction mixture was cooled, poured into a saturated aqueous solution of NH_4Cl (200 mL), and extracted with ethyl acetate (50.0 mL) three times. The combined organic layers were washed with a saturated aqueous solution of NH_4Cl (150 mL) and two times with brine (150 mL), dried over anhydrous Na_2SO_4, and concentrated under reduced pressure. Purification of the crude product by column chromatography on silica gel (eluent: hexane/EtOAc/ Et_3N (90 : 1 : 0.009) gave **S2** (1.73 g, 83%) as a yellow oil. ^{1}H NMR (400 MHz, acetone-*d*$_6$): δ = 7.61 – 7.52 (m, 3H), 7.46 – 7.39 (m, 3H), 7.33 (dd, J = 8.7 Hz, 2.4 Hz, 1H), 7.19 – 7.14 (m, 1H), 4.42 (q, *J* = 7.0 Hz, 2H), 2.48 (t, *J* = 7.0 Hz, 2H), 1.67 – 1.57 (m, 2H), 1.52 – 1.42 (m, 2H), 1.41 – 1.30 (m, 5H), 0.91 (t, *J* = 7.2 Hz, 3H). ^{13}C NMR (101 MHz, acetone-*d*$_6$): δ = 151.2, 144.9, 133.0, 131.6, 130.2, 129.2, 124.8, 122.6, 120.0, 117.9, 97.1, 78.1, 41.1, 31.9, 29.2, 22.9, 20.1, 14.3, 11.2. HRMS (ESI): *m*/*z* calcd for $C_{21}H_{24}ClN_3$+H$^+$: 354.1732 [*M*+H]$^+$; found: 354.1731.

4,6-Dibromo-3-pentylcinnoline (S3a): Cinnoline **S3a** was synthesized in accordance with the general procedure for the Richter-type cyclization from triazene **1a** (1.00 g, 2.51 mmol, 1.00

equiv). Purification of the crude product by column chromatography using hexane/EtOAc (20:1) as the eluent gave **S3a** (805 mg, 90 %) as a yellow solid. m.p. 98–99 °C. The spectral data correspond to the data reported earlier.[2] ^{1}H NMR (400 MHz, acetone-d_6) δ = 8.44 (d, *J* = 9.0 Hz, 1H), 8.38 (d, *J* = 2.1 Hz, 1H), 8.09 (dd, *J* = 9.0 Hz, 2.1 Hz, 1H), 3.46 – 3.38 (m, 2H), 2.00 – 1.87 (m, 2H), 1.55 – 1.37 (m, 4H), 0.94 (t, *J*=7.1 Hz, 3H).

4-Bromo-6-chloro-3-pentylcinnoline (S3b): Cinnoline **S3b** was synthesized in accordance with the general procedure for the Richter-type cyclization from triazene **S2** (968 mg, 2.74 mmol). Purification of the crude product by column chromatography using hexane/EtOAc (20:1 → 15:1) as the eluent gave **S3b** (704 mg, 82 %) as an orange solid. m.p. 35–36 °C. ^{1}H NMR (400 MHz, acetone-d_6): δ = 8.47 (d, *J* = 9.0 Hz, 1H), 8.12 (d, *J* = 2.2 Hz, 1H), 7.91 (dd, *J* = 9.0 Hz, 2.2 Hz, 1H), 3.40 – 3.34 (m, 2H), 1.94 – 1.85 (m, 2H), 1.49 – 1.38 (m, 4H), 0.92 (t, *J* = 7.1 Hz, 3H). ^{13}C NMR (101 MHz, acetone-d_6): δ = 158.5, 148.8, 139.3, 133.0, 132.4, 128.1, 125.7, 125.3, 36.8, 32.3, 29.3, 23.1, 14.3. HRMS (ESI): *m*/*z* calcd for $C_{13}H_{14}BrClN_2$+H^+: 315.0081 [*M*+H]$^+$; found: 315.0067.

4-Azido-6-bromo-3-pentylcinnoline (S4a): Azidocinnoline **S4a** was synthesized in accordance with the general procedure for the nucleophilic substitution from 4-bromocinnoline **S3a** (804 mg, 2.25 mmol, 1 equiv) and NaN_3 (730 mg, 11.2 mmol, 5 equiv). Purification of the crude product by column chromatography using hexane / EtOAc (10:1) as the eluent gave **S4a** (606 mg, 84 %) as a yellow solid. m.p. 78–79 °C. ^{1}H NMR (400 MHz, acetone-d_6) δ = 8.41 (d, *J* = 2.0 Hz, 1H), 8.36 (d, *J* = 9.0 Hz, 1H), 8.02 (dd, *J* = 9.0 Hz, 2.0 Hz, 1H), 3.42 – 3.35 (m, 2H), 1.99 – 1.88 (m, 2H), 1.55 – 1.38 (m, 4H), 0.94 (t, *J* = 7.1 Hz, 3H).

4-Azido-6-chloro-3-pentylcinnoline (S4b): Azidocinnoline **S4b** was synthesized in accordance with the general procedure for the nucleophilic substitution from 4-bromocinnoline **S4b** (536 mg, 1.71 mmol, 1 equiv) and NaN_3 (556 mg, 8.55 mmol, 5 equiv). Purification of the crude product by column chromatography using hexane/EtOAc (10:1) as the eluent gave **S4b** (435 mg, 92 %) as aт orange solid. m.p. 54–55 °C solid. ^{1}H NMR (400 MHz, acetone-d_6) δ = 8.41 (d, *J* = 9.1 Hz, 1H), 8.18 (d, *J* = 2.1 Hz, 1H), 7.86 (dd, *J* = 9.1 Hz, 2.1 Hz, 1H), 3.40 – 3.29 (m, 2H), 1.91 (m, 2H), 1.53 – 1.36 (m, 4H), 0.92 (t, *J* = 7.0 Hz, 3H). ^{13}C NMR (101 MHz, acetone-d_6) δ = 151.9, 149.2, 137.5, 132.6, 132.4, 132.1, 122.0, 121.3, 32.9, 32.3, 29.9, 23.1, 14.3. HRMS (ESI): *m*/*z* calcd for $C_{13}H_{14}ClN_5$+H^+: 276.1010 [*M*+H]$^+$; found: 276.1009.

6-Bromo-3-pentylcinnolin-4-amine (7a): Amine **7a** was obtained in accordance with the general procedure for the reduction from azide **S4a** (60.0 mg, 0.188 mmol) and $NaBH_4$ (11.0 mg, 0.282 mmol, 1.50 equiv) in methanol (1.88 mL). The crude product as a beige solid (46.8 mg, 85 %) was a pure and did not require additional purification. Decomp. at 260 °C. ^{1}H NMR (400 MHz, DMSO-d_6): δ = 8.57 (d, *J* = 2.0 Hz, 1H), 7.95 (d, *J* = 9.1 Hz, 1H), 7.76 (dd, *J* = 9.1 Hz, 2.0 Hz, 1H), 6.94 (s, 2H), 3.01 – 2.93 (m, 2H), 1.77 – 1.65 (m, 2H), 1.35 (m, 4H), 0.91 – 0.82 (m, 3H). ^{13}C NMR (101 MHz, DMSO-d_6): δ = 147.2, 142.8, 138.4, 132.8, 131.0, 124.7, 120.5, 116.1, 31.6, 31.4, 27.6, 22.6, 14.4. HRMS (ESI): *m*/*z* calcd for $C_{13}H_{15}BrN_3$+H^+: 294.0600 [*M*+H]$^+$; found: 294.0608.

6-Chloro-3-pentylcinnolin-4-amine (7b): Amine **7b** was obtained in accordance with the general procedure for the reduction from azide **S4b** (57.8 mg, 0.210 mmol) and $NaBH_4$ (12.2 mg, 0.320 mmol, 1.50 equiv) in methanol (2.10 mL). The crude product as a beige solid (38.7 mg, 74 %) was a pure and did not require additional purification. m.p. 213–215 °C. ^{1}H NMR (400 MHz, DMSO-d_6) δ 8.41 (d, *J* = 2.2 Hz, 1H), 8.03 (d, *J* = 9.1 Hz, 1H), 7.65 (dd, *J* = 9.1 Hz, 2.2 Hz, 1H), 6.93 (s, 2H), 3.01 – 2.93 (m, 2H), 1.71 (m, 2H), 1.35 (m, 4H), 0.90 – 0.83 (m, 3H). ^{13}C NMR (101 MHz, DMSO-d_6) δ = 146.6, 142.3, 138.1, 131.4, 130.5, 129.8, 120.9, 115.2, 31.1, 30.9, 27.1, 22.1, 14.0. HRMS (ESI): *m*/*z* calcd for $C_{13}H_{15}ClN_3$+H^+: 250.1106 [*M*+H]$^+$; found: 250.1111.

References

1. Danilkina, N.A.; Vlasov, P.S.; Vodianik, S.M.; Kruchinin, A.A.; Vlasov, Y.G.; Balova, I.A. Synthesis and chemosensing properties of cinnoline-containing poly(arylene ethynylene)s. *Beilstein J. Org. Chem.* **2015**, *11*, 373–384.

2. Danilkina, N.A.; Bukhtiiarova, N.S.; Govdi, A.I.; Vasileva, A.A.; Rumyantsev, A.M.; Volkov, A.A.; Sharaev, N.I.; Povolotskiy, A. V.; Boyarskaya, I.A.; Kornyakov, I. V.; et al. Synthesis and Properties of 6-Aryl-4-azidocinnolines and 6-Aryl-4-(1,2,3-1H-triazol-1-yl)cinnolines. *Molecules* **2019**, *24*, 2386.
